# Forest dynamics where typhoon winds blow

**DOI:** 10.1111/nph.20350

**Published:** 2024-12-14

**Authors:** Aland H. Y. Chan, Toby D. Jackson, Ying Ki Law, E‐Ping Rau, David A. Coomes

**Affiliations:** ^1^ Conservation Research Institute and Department of Plant Sciences University of Cambridge Downing St. Cambridge CB2 3EA UK; ^2^ School of Biological Sciences University of Hong Kong Pok Fu Lam Hong Kong

**Keywords:** LiDAR, plantation, rainforest, resilience, topography, tropical cyclone, typhoon, wind modelling

## Abstract

- Tropical cyclones (TCs) sporadically cause extensive damage to forests. However, little is known about how TCs affect forest dynamics in mountainous terrain, due to difficulties in modelling wind flows and quantifying structural changes. Typhoon Mangkhut (2018) was the strongest TC to strike Hong Kong in over 40 yr, with gusts > 250 km h^−1^. Remarkably, the event was captured by a dense anemometer network and repeated LiDAR surveys across natural forests and plantations.
- We mapped long‐term mean and extreme wind speeds using CFD models and analysed corresponding changes in canopy height, which uncovered TC‐forest dynamics at unprecedented scales (> 400 000 pixels, 1108 km^2^).
- Forest height was more strongly limited by wind exposure than by background topography, a limitation attributable to a dynamic equilibrium between growth and disproportionate TC damage to taller forests. Counterintuitively, wind‐sheltered forests also suffered heavy damage. As a result, canopies of wind‐sheltered forests were more rugged, which contrasted with flat‐topped forests at wind‐exposed sites. Plantations were more susceptible to TCs compared to natural rainforests of similar stature (canopy height change −0.86 m vs −0.39 m).
- Our findings highlight TCs as important, often overlooked factor that fundamentally shapes forest structure and dynamics.

## Introduction

Tropical cyclones (TCs), also known as typhoons or hurricanes, are rotating storm systems that bring strong winds and heavy rainfall, often causing substantial damage to natural ecosystems. Even TCs graded 1–2 on the five‐point Saffir–Simpson scale bring sustained wind speeds > 125 km h^−1^, leading to defoliation, branch breakage, bole snapping, and uprooting of forest trees (Tanner *et al*., 1991; Everham & Brokaw, 1996; Negrón‐Juárez *et al*., 2014; Lin *et al*., 2020). TCs cause substantial loss of aboveground forest biomass (AGB), with West Mexican and Puerto Rican forests reportedly losing 34% (Parker *et al*., 2018) and 23% (Hall *et al*., 2020) of ABG after category 3–4 TC events, respectively. TCs change forest structure, not only by damaging trees but also by remodelling tree architecture amongst survivors (Bonnesoeur *et al*., 2016; Ankori‐Karlinsky *et al*., 2024). Regions that frequently experience strong TCs have shorter forests with higher stem densities (De Gouvenain & Silander, 2003; Ibanez *et al*., 2019; Lin *et al*., 2020), with trees investing into larger basal areas relative to their heights (Ibanez *et al*., 2019). Under climate change, TCs are becoming less frequent but more intense (Kossin *et al*., 2020; Chand *et al*., 2022) and are shifting towards higher latitudes (Murakami *et al*., 2020; Chand *et al*., 2022). To predict how these changes might affect forests in the future, it is critical that we have a comprehensive understanding of wind‐forest dynamics at various spatiotemporal scales (Ennos, 1997; Lin *et al*., 2020).

We currently have limited knowledge on how wind, topography, and forest structure affect forest resistance to TCs at a landscape scale. Previous studies have shown that canopy height, soil type, stock density, and management action (e.g. thinning) could all affect forest resistance to strong winds (Cremer *et al*., 1982; Martin & Ogden, 2006; Gardiner, 2021). However, most of these studies were carried out in coniferous monocultures on flat terrain. We now know that the most valuable forests from biodiversity, carbon, and ecosystem services stand points are those with complex canopy structures (Bohn & Huth, 2016; Jucker *et al*., 2018; Zhu *et al*., 2023). Much of these forests also grow on rugged landscapes, where sites a mere few hundred meters apart could have vastly different wind regimes (Finnigan *et al*., 2020). Only a handful of studies have investigated the factors affecting TC‐resistance in these more complex systems (Boucher, 1990; Tanner *et al*., 1991; Martin & Ogden, 2006; Lin *et al*., 2020; Ni *et al*., 2021). Most of these studies are based on field observations with small sample sizes and none have explicitly modelled wind (either long term or during TCs) across the landscape. Thus, the relationship between site‐level exposure to wind and the patterns of damage remains poorly resolved. We also have very little understanding of how wind damage during TCs shape forest structure over longer time scales. At a regional level, Gorgens *et al*. (2021) found that wind affects the distribution of giant trees in the Amazon basin. Chi *et al*. (2015) suggested that typhoons reversed the elevation‐tree height gradient in Taiwan by disproportionally impacting lowland vegetation. However, to our knowledge, no studies have explored whether long‐term effects of TCs on forest height operate on finer spatial scales. In particular, it is unclear whether these wind‐effects are more important than other environmental variables, such as wetness or aspect, in shaping local forest structures.

Monitoring forest damage after TCs is no trivial task. Many existing studies are based on field measurements in established forest inventory plots, which provide detailed measurements of tree damage and mortality but only over limited spatial scales (Tanner *et al*., 1991; Everham & Brokaw, 1996). A few recent studies have turned to analysing changes in satellite multispectral imagery, but changes in vegetation indices such as the normalised difference vegetation index or enhanced vegetation index primarily reflect defoliation and are only indirectly linked to structural damage (Rossi *et al*., 2013; Abbas *et al*., 2020; Hall *et al*., 2020; Xu *et al*., 2021). The development of repeated airborne laser scanning provides a solution to this. By generating point clouds from millions of returns, light detection and ranging (LiDAR) datasets can produce detailed maps of both canopy structure and background topography across large spatial scales. Comparing repeated LiDAR scans provides unparalleled information on forest structural responses against wind. The main constraint of LiDAR is that it is expensive to collect and we cannot predict the arrival of extreme TCs. Hence, datasets rarely capture forest conditions both before and after devastating TCs.

Similarly, measuring and modelling wind across a forested, mountainous site is notoriously difficult (Finnigan *et al*., 2020). Fundamental models of wind flow across flat terrain assume that wind speeds exhibit a logarithmic height profile, depending on the roughness of the surface (Wieringa, 1986), but these models fail to capture how wind interacts with complex terrain. Wind speeds increase significantly on windward slopes but are sheltered on leeward slopes (Lemelin *et al*., 1988; Miller & Davenport, 1998; Belcher *et al*., 2011; Finnigan *et al*., 2020). The position of the wind shadow cast by mountains depends on wind direction, while the size of the wind shadow depends on wind speed and the associated deflection of wind (Belcher *et al*., 2011; Finnigan *et al*., 2020). On steeper hills, separation bubbles could form on leeward slopes, which cause wind near the boundary layer to reverse direction (Kaimal & Finnigan, 1994; Belcher *et al*., 2011; Finnigan *et al*., 2020). In narrow valleys, the Venturi effect can speed up incoming wind (Mikkola *et al*., 2023). Modelling these effects is challenging, and anemometer measurements for training and validation are often unavailable (Shah *et al*., 2023). As a result, most studies on TCs avoid modelling local wind speeds and rather resort to proxies of wind exposure, such as rainfall, aspect, elevation, or topographical exposure (TOPEX) (Wilson, 1984; Albrecht *et al*., 2019; Morimoto *et al*., 2019; Araujo *et al*., 2021; Gardiner, 2021). To our knowledge, no study has combined wind modelling with repeated LiDAR surveys to assess forest resistance to strong winds.

New datasets available for the mountainous countryside of Hong Kong provide a unique opportunity wind modelling and TC damage assessment. In September of 2018, subtropical rainforests on the rugged landscape were hit by Typhoon Mangkhut. The typhoon was the strongest TC to affect Hong Kong in over four decades, bringing 10‐min average wind speeds of >190 km h^−1^ in exposed areas (category 3 on the Saffir–Simpson scale) (Hong Kong Observatory, 2023). Remarkably, the whole area was surveyed by airborne LiDAR scans in 2010, 2017, and 2020. These LiDAR scans captured structural changes of forests through time and provide a rare opportunity to study both pretyphoon growth and post‐typhoon damage across large areas. Furthermore, hourly wind data are available from 28 non‐urban automatic weather stations scattered across the rugged terrain (Hong Kong Observatory, 2023). This allowed us to properly validate wind maps generated by computational fluid dynamics (CFD) modelling software, which estimates near‐surface wind speeds from a given digital surface model. In this study, we utilised the rare availability of repeated LiDAR and wind data to advance our understanding of how TCs affect forests on rugged terrains. In particular, we addressed five research questions:

1. How important was wind compared to other environmental variables in limiting local forest height?
2. Does the long‐term effect of strong TCs impose limits on local forest height?
3. How do forest resistance to strong TCs affect forest rugosity and structure?
4. How did the interactions between forest height, local wind regime, and background topography affect forest resistance to extreme TCs?
5. Were natural forests more resistant to extreme TCs than plantations?

## Materials and Methods

### Study area and Typhoon Mangkhut

**Fig. 1:**
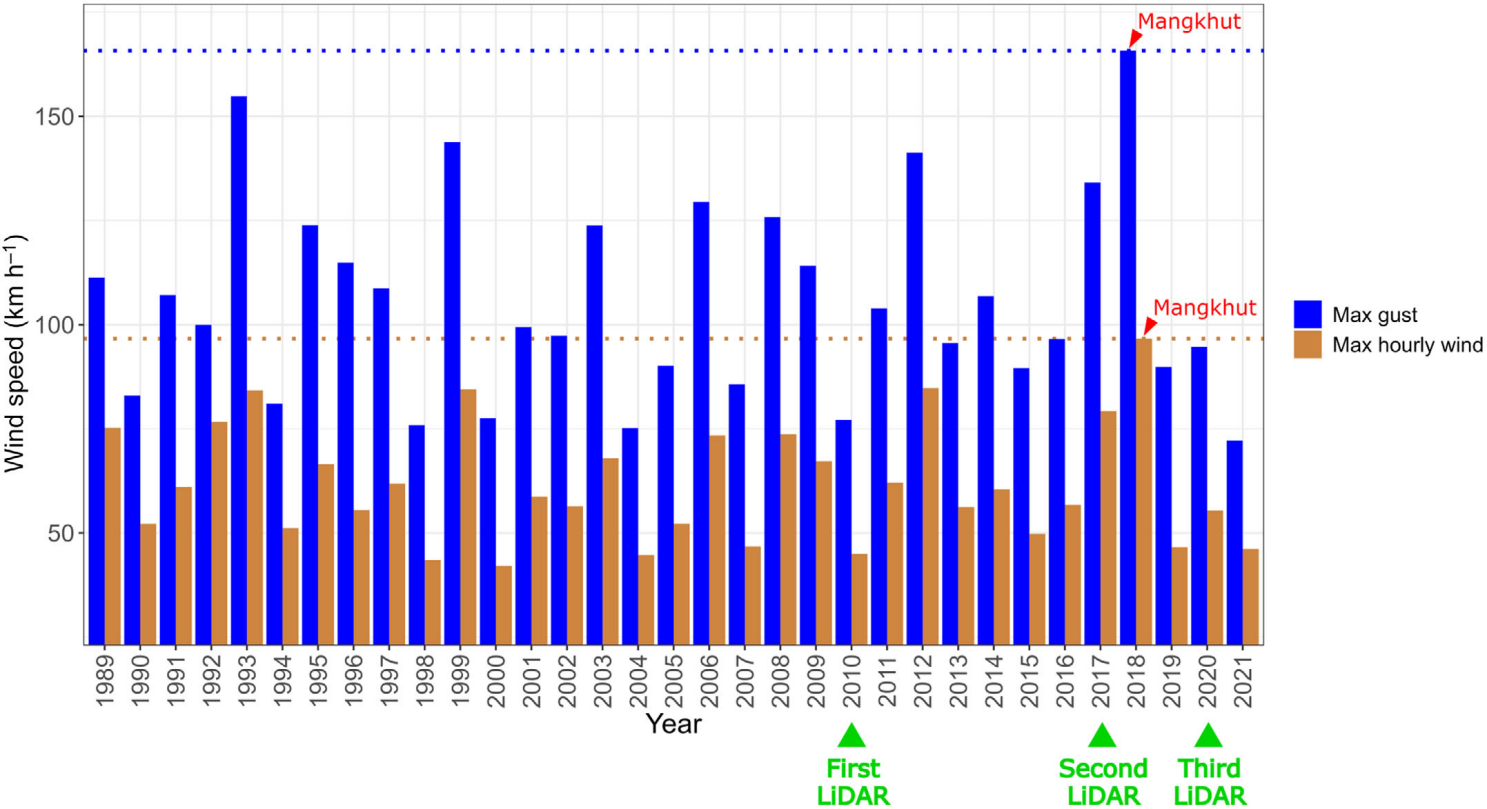
Typhoon Mangkhut is the strongest tropical cyclones (TC) that affected Hong Kong in decades. Hourly wind speeds and gusts were averaged across 28 automatic weather stations. The bars correspond to the maximum averaged hourly wind speeds and gusts each year.

Hong Kong (22°16′8″N, 113°57′6″E) has a wet subtropical climate, receiving over 2400 mm of rainfall per year with an average temperature of 23.3°C (1961–2022). Despite its reputation as a densely populated city, over 60% of the total land area (1110 km^2^) is covered with natural vegetation, with another 4% covered by tree plantations scattered across the territory. The landscape was almost devoid of forests by the close of the Second World War, but forests have subsequently recovered following widespread agricultural abandonment and better legal protection. As of 2020, the vegetated countryside was composed of a mosaic of broadleaved‐evergreen rainforests (53%), shrublands (41%) and grasslands (6%) (Abbas *et al*., 2016; Chan & Coomes, 2024). With a median slope of 0.47, the countryside of Hong Kong is rugged. Dotted across the territory are the over 300 steep‐sided hills of heights > 100 meters above sea level (m asl), with the tallest, Tai Mo Shan, rising to 957 m asl. Hong Kong lies within the west Pacific TC hotspot, experiencing multiple TCs each year (Hong Kong Observatory, 2023). Typhoon Mangkhut on the 16^th^ September, 2018 represents the strongest typhoon that affected the territory in decades (Fig. 1). Anemometers in exposed areas recorded hourly average wind speeds of > 150 km h^−1^, 10‐min average wind speeds of > 190 km h^−1^, and gusts > 250 km h^−1^ (category 3 on the Saffir–Simpson scale) (Hong Kong Observatory, 2023).

### Repeated LiDAR surveys of canopy heights, rugosity, and topography

**Fig. 2:**
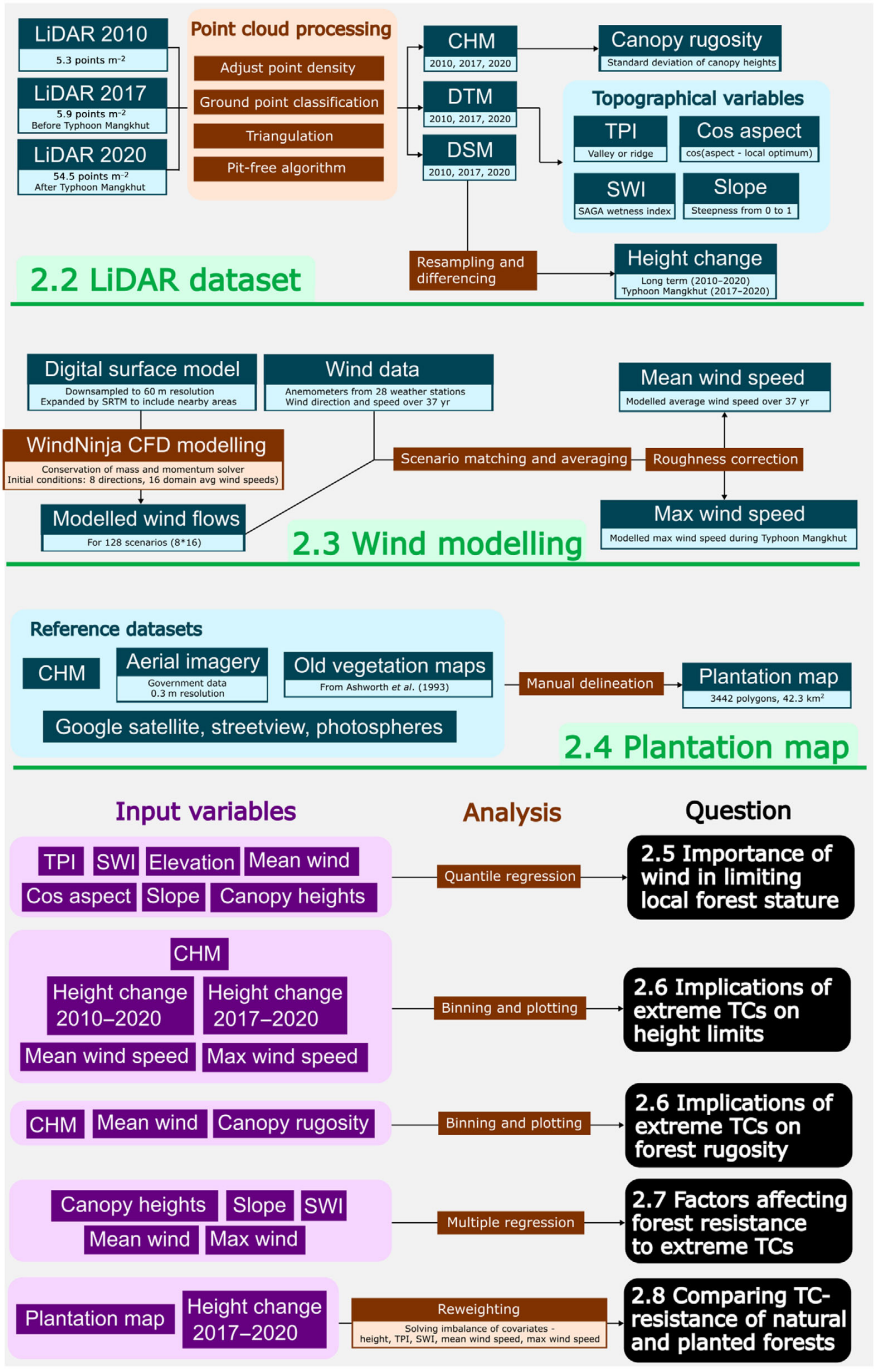
Flow chart for the methodology of the study. Numbers correspond to section numbering.

Three repeated LiDAR scans (2010, 2017, 2020) were used to reconstruct background topography and changes in forest structure. Technical specifications of the LiDAR surveys are listed in Table 1 and the overall processing workflow is summarised in Fig. 2. LiDAR point clouds were processed using *LAStools* (Isenburg, 2020). Digital terrain models (DTMs) were created by ground‐classifying LiDAR returns with *lasground_new* and triangulating ground returns with *blast2dem*. Canopy height models (CHMs) were created using the pit‐free algorithm described in Khosravipour *et al*. (2014). Finally, digital surface models (DSMs) were built using the ‐spike_free option in *las2dem* Khosravipour *et al*. (2016). The DTMs, DSMs, and CHMs were all created with an initial spatial resolution of 1 m. Canopy height changes between 2010, 2017, and 2020 were calculated by differencing the relevant DSMs. We chose to difference DSMs (absolute heights) instead of CHMs (heights relative to ground elevation) since it avoids errors in ground classification, especially in dense rainforests on steep terrain. However, DSMs are more sensitive to errors in absolute height, so we differenced the DTMs to check whether there were significant biases in absolute heights. Scan lines across the Sai Kung region in the 2017 dataset was found to have a systematic but consistent bias in absolute heights, so we isolated the flightline and corrected the bias using geodetic control points of known elevations (HK Lands Department, 2019). Lastly, we addressed issues related to man‐made objects (Supporting Information Notes S1) and point density differences (Notes S2; Figs S1–S3) to ensure comparability between scans.

**Table 1:** Technical specifications of the three LiDAR datasets in Hong Kong. Typhoon Mangkhut affected the territory on the 16^th^ September 2018.

Dataset	2010	2017	2020
Date acquired	December 2010–January 2011	November 2017	December 2019–February 2020
Coverage	Whole territory	500 km^2^	Whole territory
Carrier	Manned aircraft	Manned aircraft	Helicopter
Scanner	Optech Gimini ALTM	RIEGL LMS‐Q780	–
Flight height	1000–1200 m	2147–2893 m	600 m
Point density	5.3 points m^−2^	5.9 points m^−2^	54.5 points m^−2^
Returns per pulse	4 returns	Up to 7 returns	8 returns

In addition to canopy height, we estimated canopy rugosity in 2010 and 2020 by calculating the SD of canopy height at 30 m spatial resolution. SD of height was chosen as the metric for canopy rugosity due to its ease of interpretation and stability across pixels with low‐canopy heights.

We also generated rasters for four topographical variables – slope, aspect, topographic position index (TPI), and SAGA wetness index (SWI) – following methods described in Chan & Coomes (2024). Aspect, which is a cyclical variable, was linearised by subtracting the reported local optimal aspect for forests (5.795 radians) and taking the cosine (cos_aspect = cos(aspect – 5.795)) (Chan & Coomes, 2024). TPI is a variable calculated from the DTM that refers to whether the site sits in valleys (low TPI) or on ridges (high TPI). SWI, also calculated from the DTM, refers to the catchment area of a pixel in question (i.e. how much water flows to the pixel) (Mattivi *et al*., 2019; Chan & Coomes, 2024). The resulting rasters with different resolutions – elevation (1 m), slope (1 m), aspect (30 m), TPI (15 m), SWI (15 m) – were down‐sampled to 30 m resolution.

### Wind modelling

The aim of the wind modelling was to obtain two maps, one showing the mean long‐term wind speed across Hong Kong and one showing maximum wind speeds during Typhoon Mangkhut. To achieve this, we used an advance wind flow model that made predictions based on 37 yr of wind data and a high‐resolution topographic map (Fig. 2).

Wind data were collected by the Hong Kong Observatory across 28 nonurban automatic weather stations (Fig. S4), with records spanning 37 yr (1984–2022). In each station, the wind speed, direction, and gust were continuously measured at 10 m aboveground and were averaged across hourly time steps.

We used WindNinja to carry out computational fluid dynamics (CFD) modelling. WindNinja (available on https://weather.firelab.org/windninja/) is a software that estimates wind speed and direction based on a digital surface model (DSM) and a set of initial conditions. While the software is computationally limited in resolving wind flows at resolutions < 50 m, the ‘conservation of mass and momentum solver’ in WindNinja create realistic wind maps at landscape scales on both windward and leeward slopes by building on the capabilities of OpenFOAM (Weller *et al*., 1998; Forthofer *et al*., 2014). We ran the model on the digital surface model of the study area (‘Repeated LiDAR surveys of canopy heights, rugosity, and topography’ in the Materials and Methods section) for combinations of 8 compass directions and 16 wind speeds, generating speed and direction rasters for a total of 128 scenarios. Based on the observed wind directions and speeds, we then took combinations of these scenarios to create wall‐to‐wall wind maps, including one that represented long‐term mean wind speed between 1984–2022 and another for maximum wind speed between 2017–2020 (mainly driven by Typhoon Mangkhut). Due to computational constraints, wind flows were modelled at 60 m ground resolution, with rasters then resampled to 30 m resolution for analysis. A more detailed description of the wind modelling pipeline and results from the cross validation exercise (Figs S5, S6; Table S1) can be found in Notes S3 (Wieringa, 1986).

### Vegetation and plantation maps

We gathered a vegetation map time series of the study area to focus our investigation on forests. The maps were generated by classifying Landsat composites into five classes (forest, shrubland, grassland, water, and nonvegetation) using a supervised random forest (RF) model. Technical details of the vegetation maps can be found in Chan & Coomes (2024).

**Fig. 3:**
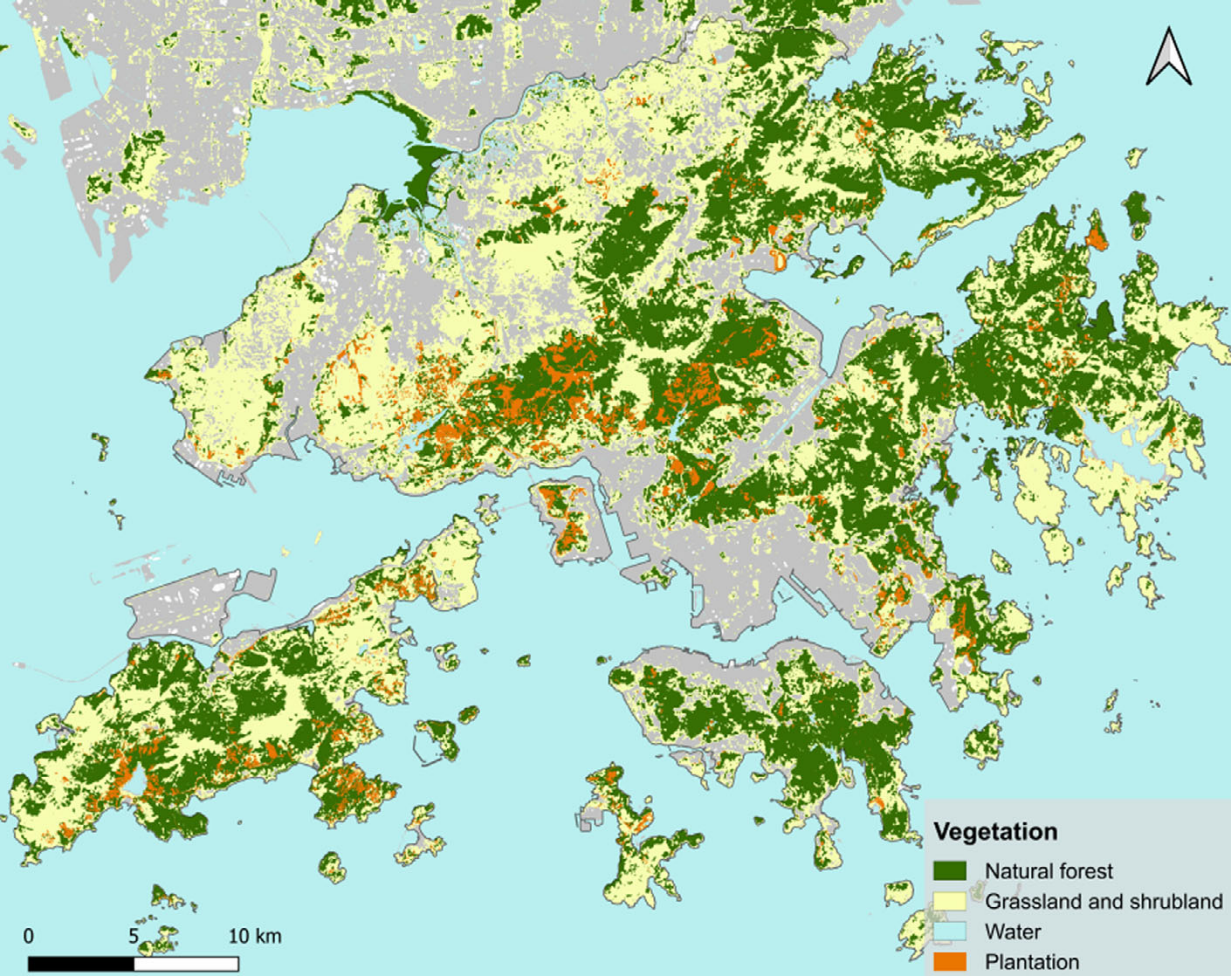
Distribution of natural subtropical rainforests and plantations in the study area of Hong Kong. Plantations are manually delineated with reference to Google Satellite, Google Streetview, LiDAR canopy height models (CHMs), photospheres, and Ashworth *et al*. (1993). Natural forests, shrublands, grasslands, and water bodies are based on a Landsat‐based vegetation map published in Chan & Coomes (2024).

To evaluate differences in the response of plantations and natural forests to typhoons, we created a plantation database by manual delineation. We identified plantations by visually inspecting LiDAR‐derived CHMs and aerial photos collected in 2014 (0.3 m ground resolution) (HK Lands Department, 2019). Google Satellite, Streetview, and photospheres were also widely available across the countryside of Hong Kong and provided additional ways to check the species composition of various forest stands. (Ashworth *et al*., 1993) The final plantation map contains 3442 polygons covering an area of 42.3 km^2^ (Fig. 3).

### The importance of wind in limiting local forest stature

We used quantile regression to evaluate whether these wind‐dependant limits on local forest heights were important compared to limits posed by other environmental variables. Quantile regression is a statistical technique used to model how predictors affect the position of conditional quantiles (e.g. the 25^th^, 50^th^, 95^th^ quantiles) of the response variable. It provides more flexibility than ordinary least squares regression, which focuses only on the mean. In particular, regression through the top quantiles represents a powerful tool to study limiting factors in ecology due to its robustness against other measurable or unmeasurable confounding factors (Cade *et al*., 1999; Cade & Noon, 2003; Coomes & Allen, 2007). In this study, we performed quantile regression through the 97.5^th^ percentile of 2010 and 2020 canopy heights at 30 m spatial resolution using six environmental variables as predictors, namely (1) mean wind speed, (2) elevation, (3) cosine aspect, (4) slope, (5) wetness (SWI), and (6) topographical position (TPI). Plantations were excluded as exotic tree species (e.g. *Lophostemon confertus* and *Eucalyptus* sp.) tend to have significantly different vertical profiles. Second‐order polynomials were fitted through the data as several factors had nonlinear effects on maximum canopy height. We used the results to evaluate the importance of wind limits on local forest heights compared to the other variables.

### The implications of extreme TCs on forest height limits and structure

We then explored how extreme TCs might have contributed to local forest height limits. To summarise the changes in height across a large number of pixels (*n*_2017_ = 191 744; *n*_2020_ = 406 482), we binned the data into 816 bins according to mean wind speed of the site and canopy height. Bins with small sample sizes (*n* < 10) were filtered out. For each bin, the effects of Typhoon Mangkut were visualised by calculating the changes in canopy height between 2017 and 2020. Similarly, the long‐term changes in forest stature were visualised by calculating the mean canopy height change within each bin between 2010 and 2020.

Furthermore, we investigated how the observed wind‐forest dynamics affected forest rugosity. We focused on continuous forests by removing pixels close to edges (< 45 m) or have extreme SD (SD > 10 m). The remaining dataset was still large (*n*_2010_ = 198 267; *n*_2020_ = 264 973), so we binned the data by canopy height and mean wind speed (bin size = 150 pixels) for better visualisation. We calculated the mean rugosity in each bin and created separate plots for 2010 and 2020.

### Factors affecting natural forest resistance to typhoons

We investigated how factors other than height affected forest resistance to extreme TCs using a multiple regression model. The response variable of the model was forest damage during Typhoon Mangkhut, measured as the canopy height change between 2017 and 2020. The predictors were wind, forest, and topographical variables. Recognising that multicollinearity amongst predictors could undermine the results by inflating or even flipping the signs of coefficients, we selected predictor variables with care. First, maximum wind speed during Typhoon Mangkhut correlated with long‐term mean wind speed (*R* ^2^ = 0.9, Fig. S7). To specifically identify regions that are disproportionately exposed to Typhoon Mangkhut, we transformed the variable to ‘normalised maximum wind speed’ by subtracting a multiple of the mean wind speed from the maximum wind speed. After the transformation, the variable represented whether the site was disproportionately exposed to Typhoon Mangkhut and was less dependent on mean wind speed (*R* ^2^ = 0.32, Fig. S7). Second, SWI, TPI, and slope were moderately correlated (Fig. S7). We dropped TPI as a predictor as the variable had a small effect size by itself but significantly affected the coefficients of SWI and slope when included (Fig. S7). The final model contained five predictor variables, namely (1) 2017 canopy height, (2) long‐term mean wind speed, (3) normalised maximum wind during Mangkhut, (4) wetness (SWI), and (5) slope. Lastly, to better understand how the two wind variables and forest height interacted and shaped patterns of forest damage, we included the two‐way interaction terms between (1), (2), and (3). All predictor variables were scaled by subtracting the values by the mean and dividing them by the SD. This ensured that the model produced comparable coefficients that represented the relative effect sizes of the variables (Table S2). A discussion on the potential effects of spatial autocorrelation and relevant diagnostic plots (Figs S8, S9) for the model can be found in the Notes S4.

### Comparing TC‐resistance of natural forests and plantations

Lastly, an important question in tropical rainforest restoration is whether plantations and natural forests respond differently to extreme TCs. To address this, we started by simply calculating the 2017–2020 height change and comparing the results between natural forests and plantations. This gave a holistic overview of typhoon‐related damage amongst the two forest types. The problem with this approach is that the results could be confounded by covariate imbalances. For instance, it is reasonable to expect plantations to suffer more damage simply because they were taller or disproportionally planted on exposed ridges for erosion control. To investigate whether the structure of natural forests was inherently more wind resilient than plantations after accounting for these differences, we repeated our analysis after reweighting.

Akin to pixel matching, reweighting is a statistical technique commonly used in medical research (Matschinger *et al*., 2020; Markoulidakis *et al*., 2022). It tackles covariate imbalance by assigning weights to each datapoint such that the weighted dataset has comparable covariate distributions across the categories of interest. In our case, the goal is to assign weights to pixels such that weighted natural forest pixels were comparable to plantations in terms of (1) height, (2) TPI, (3) SWI, (4) mean wind speed, and (5) maximum wind speed during the typhoon. By doing so, we can isolate the effect of forest type on TC‐resistance. The detailed reweighting methodology is based on Markoulidakis *et al*. (2022) and can be found in the Notes S5, with diagnostic data presented in Fig. S10 and Table S3.

## Results

### Mean and maximum wind maps

**Fig. 4:**
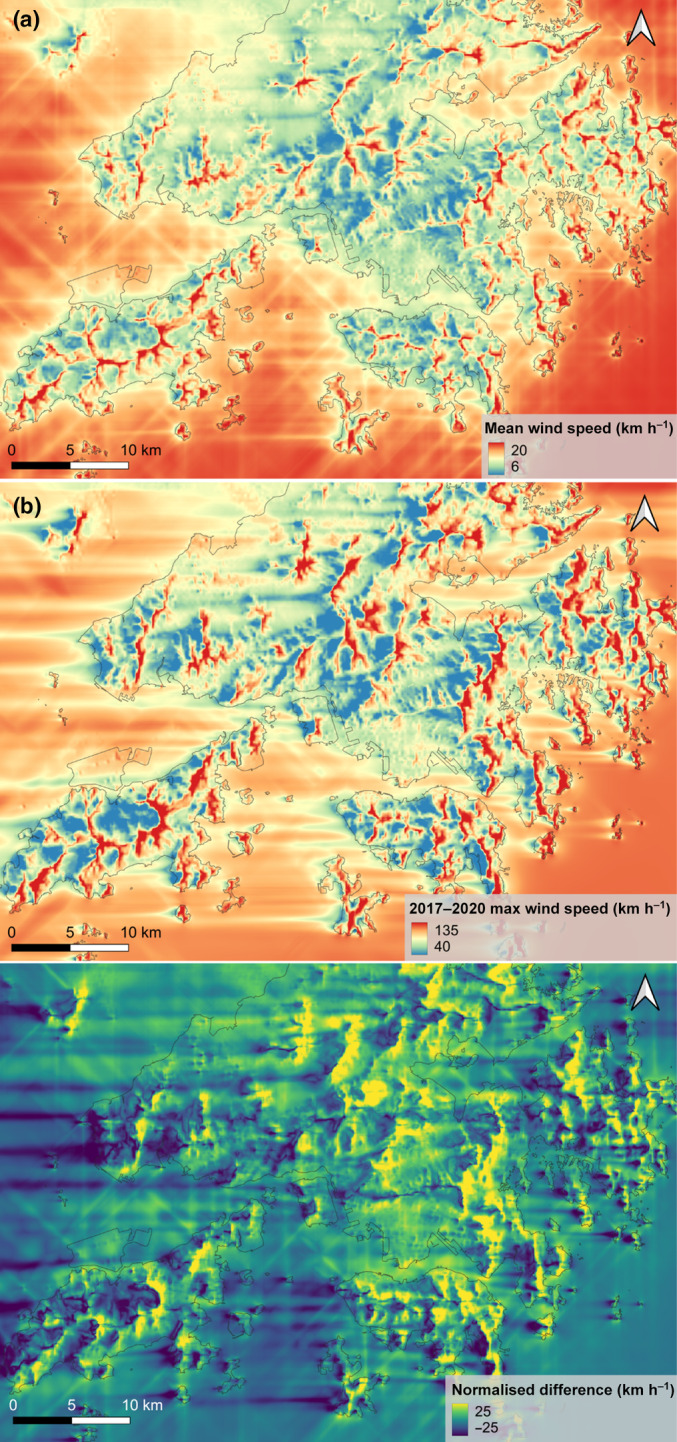
Modelled (a) long‐term mean wind speed, (b) maximum wind speed during Typhoon Mangkhut, and (c) the normalised difference between the two. The grey lines represent the outline of the terrestrial areas of Hong Kong.

The rugged topography of Hong Kong created variable wind regimes across the landscape. Overall, our CFD models outperformed null models in estimating wind flows through the rugged study area (Fig. S5), especially in high‐wind conditions (Fig. S6). Both the modelled long‐term mean wind speed and 2017–2020 maximum wind speed (Typhoon Mangkhut) showed over threefold differences across pixels. Overall, exposed sites such as ridges and mountaintops with higher long‐term mean wind speed also experienced higher wind speeds during Typhoon Mangkhut (Fig. 4). However, the typhoon brought disproportionally strong winds from the east and created prominent wind shadows towards the western slopes of mountains (Fig. 4). Therefore, there was considerable variation in maximum wind speed even amongst pixels with similar long‐term wind regimes, which can be visualised by normalising the maximum Typhoon Mangkhut wind speed raster by the long‐term mean wind speed raster (Fig. 4c).

### Forest height was limited by local wind regime

**Fig. 5:**
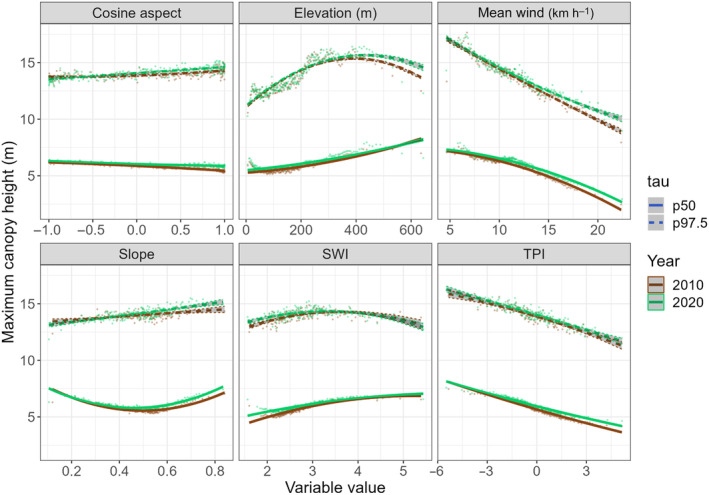
Local wind speed strongly limits canopy height. Each point represents the maximum average canopy height (97.5^th^ percentile) amongst 2000 30 m × 30 m pixels in year 2010 (brown, *n* = 324 186) or year 2020 (green, *n* = 404 612). The lines represent second order 97.5^th^ quantile regression lines. The slope is represented by a unitless ratio between 0 (flat) and 1 (vertical). SWI, SAGA wetness index; TPI, topographical position index.

Quantile regression revealed that the maximum heights of forests (97.5^th^ quantile) were strongly negatively correlated with mean wind speed. The tallest forests in the least windy sites were *c*. 50% taller than those in the windiest sites (Fig. 5). Topographical position (TPI) was the second most important variable limiting forest height, with forests in valleys (low TPI) having a higher height limit than ridges (high TPI). These patterns were not driven by collinearities with the other variables studied. Wetness (SWI), aspect, and slope all had relatively weak effects on maximum height (Fig. 3). Elevation and associated temperature regimes were also not responsible for the wind‐height relationship. Forests at higher elevations reached greater maximum heights despite having higher wind speeds and cooler temperatures (Fig. 5). Overall, the maximum forest heights (97.5^th^ percentile) remained largely unchanged between 2010 to 2020, with this height limit being more strongly correlated to local wind conditions than with other studied environmental variables.

### Forest height limits represent a dynamic equilibrium created by low‐TC resistance of tall forests

**Fig. 6:**
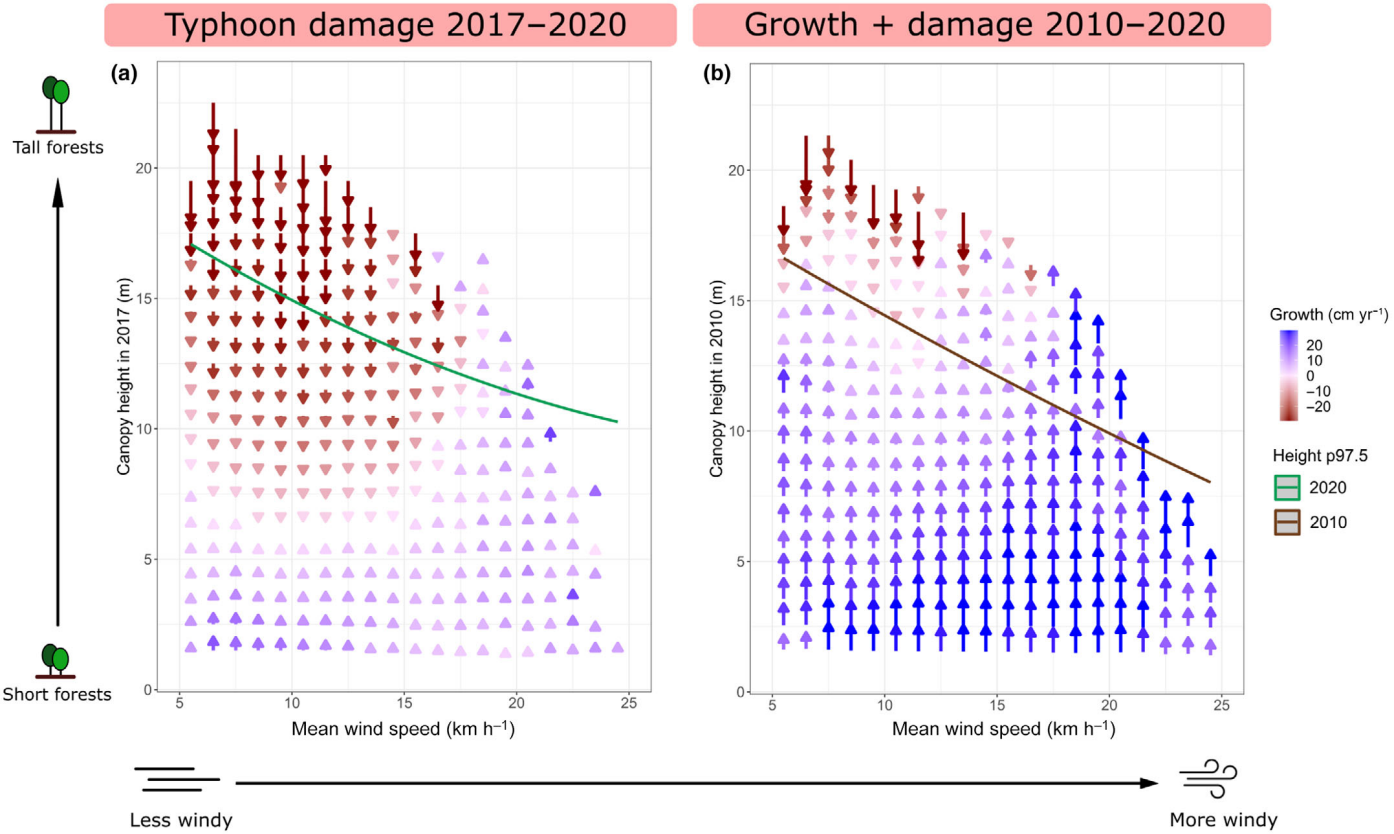
The change in canopy height (a) during the period affected by Typhoon Mangkhut (2017–2020, *n* = 191 747) and (b) over the entire study period (2010–2020, *n* = 325 511). The length of the arrows represents total change in height over the relevant time period. The colour shows the rate of height change in cm yr^−1^. The mean wind speed corresponds to modelled long‐term wind speeds over more than three decades. The lines represent the maximum canopy height (97.5^th^ percentile) estimated by second‐order quantile regression carried over from Fig. 5.

Wind limits on local forest heights were largely attributable to the vulnerability of tall forests to extreme TCs. Under the influence of Typhoon Mangkhut, taller forests (> 15 m) recorded substantial loss in height (−27.9 cm yr^−1^) between 2017–2020, while shorter forests (< 7 m) maintained growth (+3.1 cm yr^−1^) over the same period (Fig. 6a). The trend of taller forests being more wind‐susceptible was robust across a range of different maximum wind speeds (Fig. 6a). Over a longer time frame (2010–2020), growth in years without strong TCs largely offset the damage caused by extreme TCs (blue arrows, Fig. 6b). However, growth failed to offset damage once the forests get too tall for their local wind regime, creating a dynamic equilibrium that settles on the height limits shown in Fig. 5 (brown line, Fig. 6b).

### Wind‐sheltered forests had rugged structures due to higher vulnerability to tropical cyclones

**Fig. 7:**
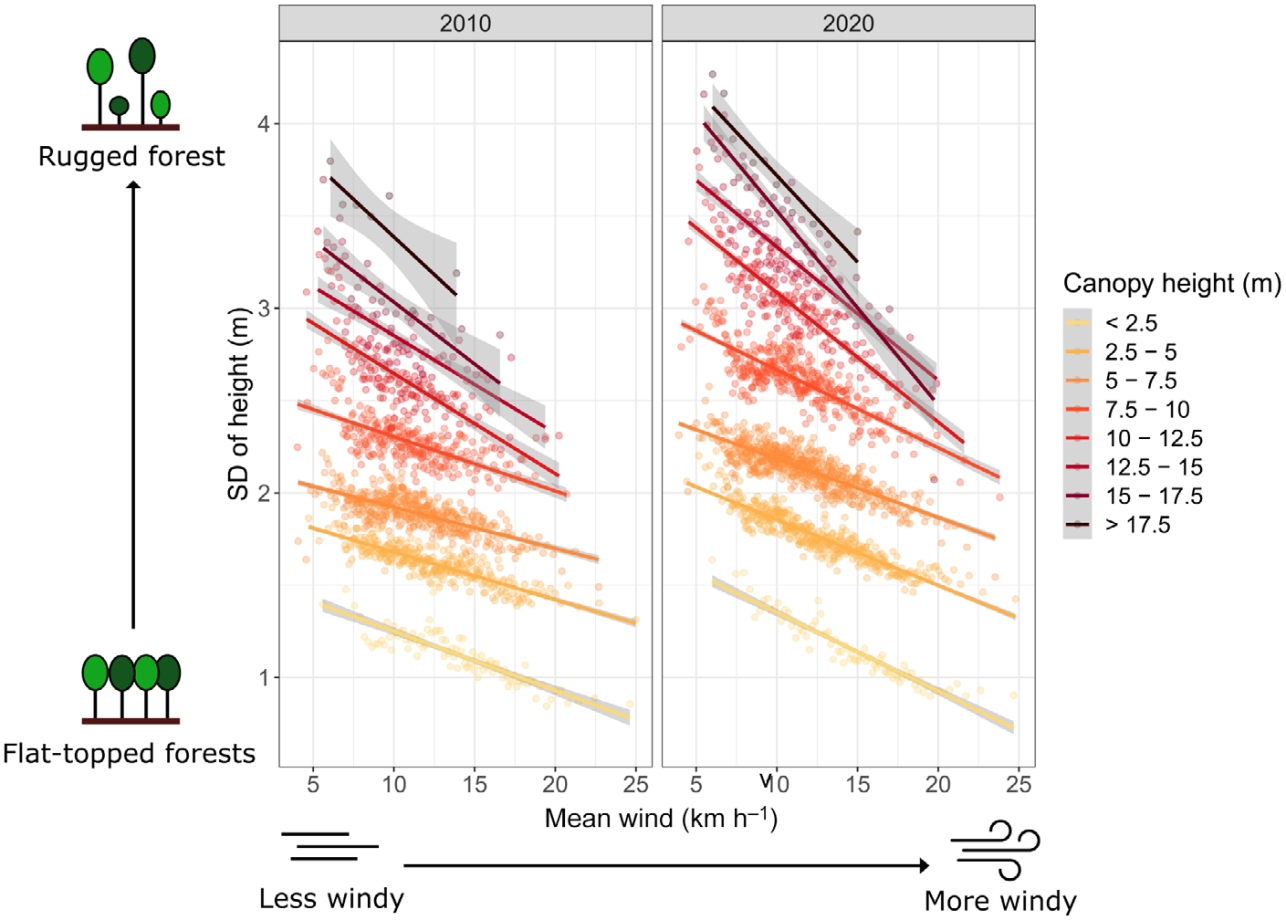
Forest rugosity against mean wind speed in 2010 (not after strong TCs) and 2020 (after Typhoon Mangkhut). Forest rugosity is measured by the SD in canopy height (m). Each point represents 150 binned pixels (*n*_2010_ = 198 267; *n*_2020_ = 264 973). Lines were created by linear regression through points in each height class.

Forests had two alternative strategies in response to wind. Forests in sheltered sites tend to grow past the local height limit (Fig. 6b) then suffer heavy damages during extreme TCs (Fig. 6a). This strategy produced rugged forests with many gaps. Amongst forest stands of comparable stature, those in sheltered sites (mean wind speed < 10 km h^−1^) had the highest SD in canopy height in both 2010 and 2020 (Fig. 7). The rugosity of these wind‐sheltered forests was also markedly higher in 2020 compared to 2010, highlighting the role of extreme TCs in producing the jagged forest structure. By contrast, forests in windy sites were wind‐acclimated and highly resistant to extreme TCs (Fig. 6a). This strategy created flat‐topped forests with lower SD in canopy height (Fig. 7). These forests were also less responsive to extreme TCs, with rugosity of wind‐exposed forests (mean wind speed > 15 km h^−1^) barely changing between 2010 and 2020 despite being hit by Typhoon Mangkhut (Fig. 7).

### Forest TC‐resistance shaped by interactions between forest structure, wind, and topography

**Fig. 8:**
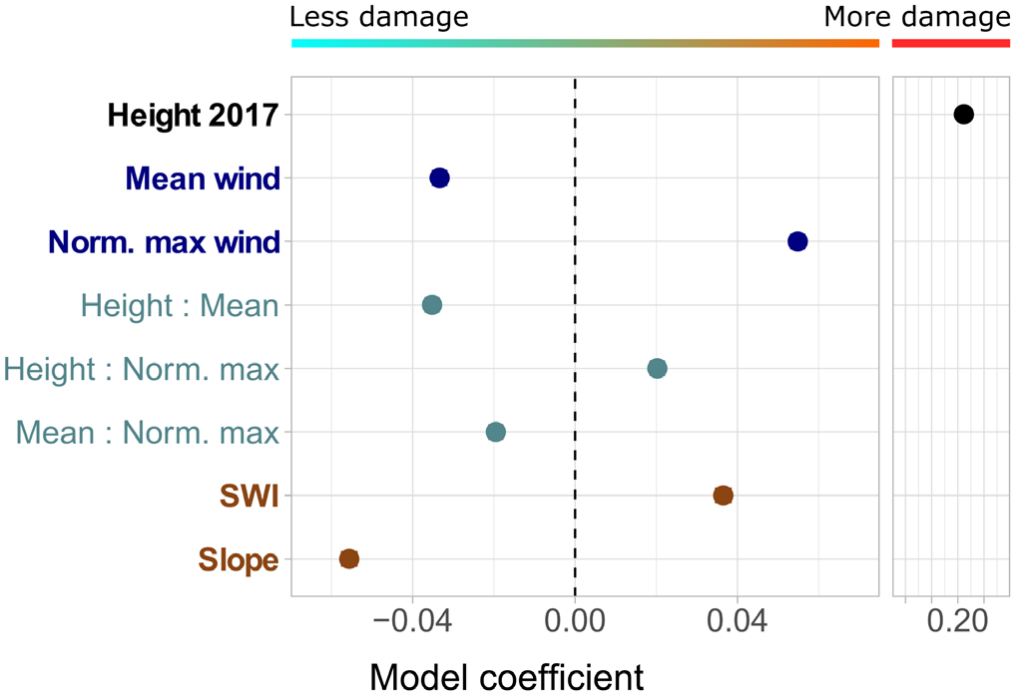
Coefficients of multiple regression model predicting damage after Typhoon Mangkhut (measured as drop in canopy height between 2017 and 2020). Norm. max wind is a variable created by normalising the maximum modelled wind speed during Typhoon Mangkhut with the long‐term mean wind speed and reflects whether the site was disproportionally affected by the event. The variables were scaled such that effect sizes and directions are comparable. Error bars (barely visible) are the SE of the coefficient estimate. SWI, Saga Wetness Index.

The multiple regression model on 2017–2020 canopy height change further revealed how forest stature, wind, and topography shaped the forest resistance to extreme tropical cyclones. Corroborating with the results in Fig. 6, forest stature was by far the strongest factor affecting TC‐resistance, with taller forests being more heavily damaged by Typhoon Mangkhut (Height 2017, Fig. 8). The model also highlighted the effects of wind acclimation as seen in Fig. 6, with wind‐sheltered forests being more heavily affected by the typhoon (Fig. 8). The model further revealed that such acclimation effect was especially pronounced in taller, more mature forests (height: mean wind, Fig. 8). As one might expect, forests that experienced disproportionately strong wind during Typhoon Mangkhut (i.e. stronger than predicted by long‐term mean wind) were more heavily damaged (norm. max, Fig. 8). Interestingly, forests appeared to have overacclimated to their long‐term wind regime, which means that forests at sites with higher long‐term mean wind speeds were less sensitive to disproportionally strong maximum wind speeds during Typhoon Mangkhut (mean: norm. max, Fig. 8). Finally, we identified two topographical factors largely orthogonal to the wind variables (Fig. S7) that had substantial effects on forest TC‐resistance – wetter sites were more susceptible to tropical cyclones (SWI, Fig. 8), while steeper sites were more resistant to damage (slope, Fig. 8).

### Plantations were more susceptible to tropical cyclones than natural forests

**Fig. 9:**
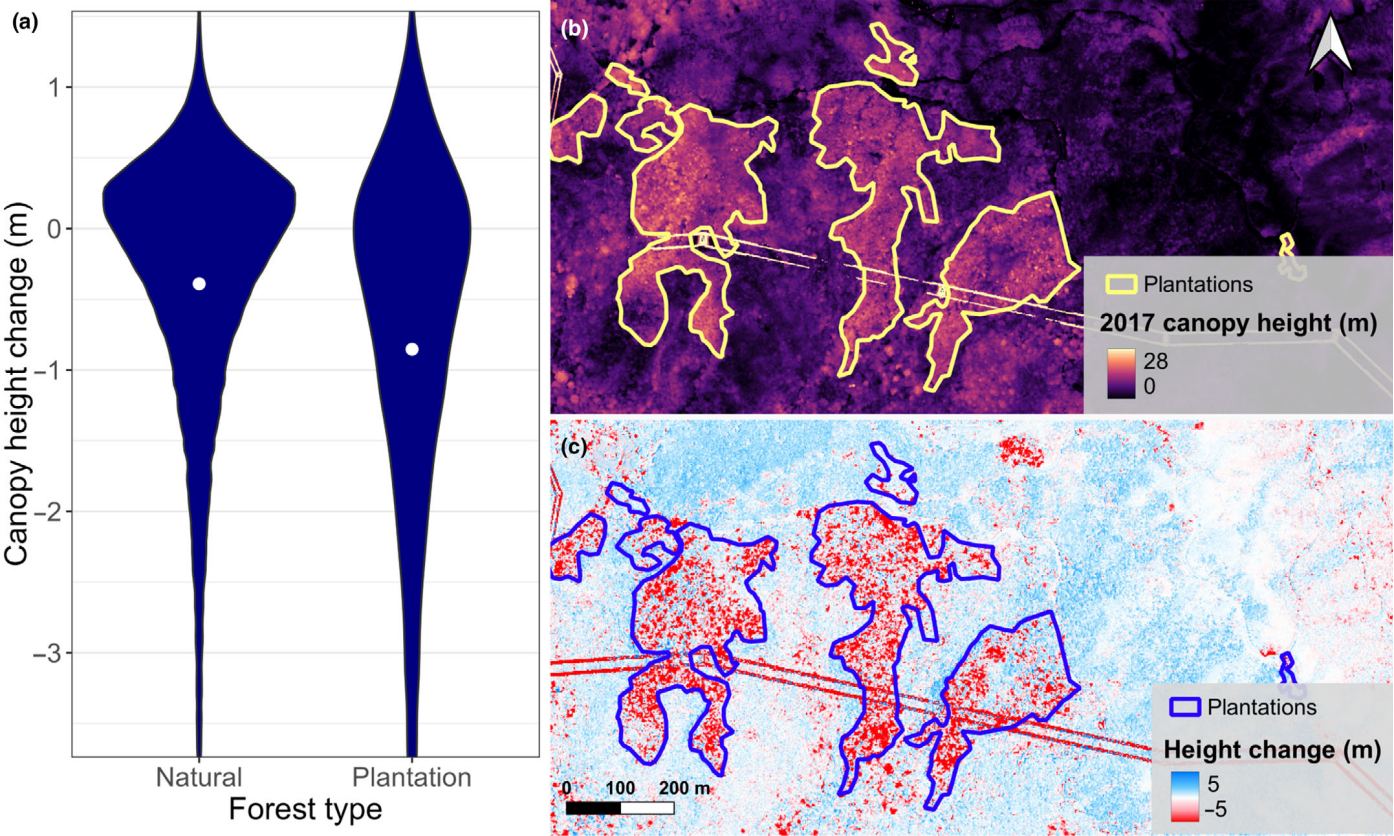
Plantations suffered heavier losses during Typhoon Mangkhut. Panel (a) contains a violin plot of height changes between 2017 and 2020. The pixels were reweighted such that the natural forests had comparable height, TPI, SWI, and wind distribution as the plantations. Effective sample sizes (ESS) were 114 838 for natural forests and 16 381 for plantations. The white dot indicates the mean of the dataset. Panel (b) shows the canopy heights of forests near Tate's Cairn, Hong Kong in 2017. Panel (c) shows the height changes of the same region between 2017 and 2020. The linear features in (b, c) are power lines, which were masked out before data analysis.

Compared to natural forests, plantations were more heavily hit by Typhoon Mangkhut. On average, plantations lost 0.86 m in height between 2017 and 2020, equivalent to 45% of the growth in the previous 7 yr. By contrast, natural forests only lost 0.1 m in height between 2017 and 2020, or 10% of the growth between 2010 and 2017. Part of these differences could be attributed to the imbalance of covariates, such as plantations being taller and being disproportionally planted on ridges (Table S3). Nevertheless, after accounting for these differences by reweighting, we found that plantations were still more than twice as susceptible to tropical cyclones (−0.86 m) compared to natural forests of similar heights and topographical positions (−0.39 m) (Fig. 9a). In particular, a larger proportion of trees were either snapped or uprooted in plantations, creating a fat tail in the violin plot of 2017–2020 height change (Fig. 9a). Visually assessing the relevant rasters revealed how entire stands of planted trees were wiped out by the typhoon (Fig. 9b,c). The scale of damage seen in these sites was not observed in natural forests in the same region (Fig. 9b,c).

## Discussion

### High‐tropical cyclone resistance of natural forests in Hong Kong

Natural forests in Hong Kong were surprisingly resistant to Typhoon Mangkhut. By summarising the changes across > 190 000 pixels we found that even the tallest (> 15 m) natural forests lost < 5% of their height between 2017 and 2020. This contrasts with reports of forests losing 39% (7.1 m) of mean canopy height and 23% of aboveground biomass (AGB) after category 4 Hurricane Maria hit Puerto Rico (Hall *et al*., 2020; Leitold *et al*., 2022). Similarly, an even larger loss in AGB (33.7%) was reported after category 4 Hurricane Patricia swept through forests in western Mexico (Parker *et al*., 2018). Instead, our findings are more in line with studies conducted in the west Pacific. Mabry *et al*. (1998) reported a 1.4% forest mortality over seven transects within Fu‐Shan Experimental Forest in Taiwan after a category 3 typhoon, while Yap *et al*. (2016) estimated a 2.64% loss in aboveground biomass after Category 4 Typhoon Imbudo hit Palanan, Philippines. These regional differences may be attributable to the higher TC frequency and therefore better wind acclimation of forests in the west Pacific typhoon hotspot (Hogan *et al*., 2018; Lin *et al*., 2020).

Plantations were much more susceptible to strong TCs, losing 0.86 m in height between 2017 and 2020. Two previous studies in the region, both based on satellite multispectral data, reported larger reductions of greenness in plantations after typhoons compared to natural forests (Abbas *et al*., 2020; Stas *et al*., 2023). By analysing the 16 381 plantation pixels covered by the extensive LiDAR dataset, we showed that the damage plantations suffered was structural and not only caused by heavier defoliation (Fig. 9).

The high susceptibility of plantations to wind damage is likely due to differences in tree architecture (Tanner *et al*., 1991; Jackson *et al*., 2019). Under higher stocking densities, trees tend to maintain height growth at the expense of diameter growth, leading to slender allometries (Cremer *et al*., 1982; Locatelli *et al*., 2016). Furthermore, trees in dense canopies tend to be sheltered and poorly wind acclimated (Cremer *et al*., 1982; Bonnesoeur *et al*., 2016). While sheltering by neighbours protects trees from wind damage in normal circumstances, extreme TCs create gaps in the canopy, which exposes previously sheltered trees to the full force of the wind (Locatelli *et al*., 2016). Several studies have found that wind damage can propagate quickly in even‐aged monocultures where trees have similar critical wind speeds (i.e. the wind speed that causes bole snapping or uprooting) (Dupont *et al*., 2015; Kamimura *et al*., 2019; Gardiner, 2021). When Typhoon Mangkhut hit Hong Kong, wind damage propagated through several planted stands and led to stand‐replacing level of damage in some sites (Fig. 9). The same patterns of damage propagation were not observed in mixed‐species broadleaved rainforest, probably due to large variations in critical wind speeds across trees of different species and age classes (Jackson *et al*., 2019; Uriarte *et al*., 2019).

Finally, it is important to recognise that not all plantations are the same. In recent years, Hong Kong gradually pivoted towards using native trees to create mixed‐species plantations. The shift was mainly biodiversity‐motivated, but mixed species stands with more complex vertical structures were also found to be more stable under strong winds (Gardiner, 2021; Gardiner *et al*., 2005; Jactel *et al*., 2017; but also see Tanner & Bellingham, 2006). Management practices such as the thinning of dense plantations can also alter wind resistance of forests by increasing wind susceptibility in the short term while fortifying the stand in the medium to long term (Cremer *et al*., 1982; Leverkus *et al*., 2021; Costa *et al*., 2023). Overall, plantations were less wind‐resistant compared to natural forests due to structural weaknesses, but mixed‐species native plantations could potentially fortify planted stands against future TCs.

### Taller forests were more susceptible to extreme tropical cyclones

Amongst natural forests, canopy height before Typhoon Manghkut was the strongest predictor of wind damage. In monocultures, foresters have long recognised that taller stands had lower critical wind speeds compared to shorter ones (Cremer *et al*., 1982; Morimoto *et al*., 2019; Gardiner, 2021; Costa *et al*., 2023). The evidence is, however, far less conclusive in structurally complex natural rainforests due to the lack of direct height measurements (Tanner *et al*., 1991; Sánchez Sánchez & Islebe, 1999; Martin & Ogden, 2006; Halder *et al*., 2021; Ni *et al*., 2021). Halder *et al*. (2021) focused on DBH measurements in the study on TC‐resistance of mangrove trees in the Sundarbans. Sánchez Sánchez & Islebe (1999) reported that small understory trees also suffered from heavy damage. Leitold *et al*. (2022) studied impacts of Hurricane Maria and found larger height losses amongst taller lowland forests compared to shorter montane forests. Ni *et al*. (2021) studied the effects of Typhoon Mangkhut in neighbouring Dinghushan and concluded that mature forests were more heavily affected compared to younger secondary forests. The patterns reported in Ni *et al*. (2021) were likely related to forest stature, but the study made no explicit measurements of tree height. By summarising results derived from > 190 000 pixels (30 m by 30 m in size), our study provided clear empirical evidence supporting taller natural forests being more susceptible to TCs, with effect size over twice that of the next most important variable studied.

### Forest height limited by a dynamic equilibrium shaped by wind and tropical cyclones

The low‐wind resistance of tall forests results in a dynamic equilibrium that constrains forest height in TC‐prone regions. In Hong Kong, much of the landscape is covered by secondary forests still moving through the successional stages (Abbas *et al*., 2016). Hence, when averaged across longer periods of time, the growth of forests largely offset damage incurred during strong typhoons. This was, however, not the case for the tallest forests that were reaching the height limits of their site‐specific wind regimes (Fig. 6b). Forests overshooting these limits were trimmed back by extreme typhoons, creating a dynamic equilibrium approximately centred around the 97.5^th^ percentile of forest height (Figs 5, 6b). These height limits are likely further shaped by the known pattern of slower height growth with respect to radial growth in sites with very strong winds (Wadsworth, 1959; Telewski & Jaffe, 1986; Thomas *et al*., 2015; Coomes *et al*., 2018; Wang *et al*., 2022). Overall, topographical wind exposure more strongly constrained forest height than by other environmental variables studied. With the wind‐driven dynamic equilibrium settling at modest heights (8–17 m), forests would rarely be limited by hydraulic (Ryan & Yoder, 1997; Gorgens *et al*., 2019; Fernández‐de‐Uña *et al*., 2023), temperature (Saremi *et al*., 2014; Chi *et al*., 2015), or nutrient (Gower *et al*., 1996) constrains. Hence, the local wind regime largely displaced other factors in limiting canopy height in TC‐prone regions (Uriarte *et al*., 2023). Interestingly, topographical position (TPI) also had clear unidirectional, albeit smaller, effects on maximum canopy height. Since topographical wetness (SWI), which better represents nutrient and water availability, had relatively minor effects on maximum canopy height, we speculate that the effects of TPI might also be wind‐related. Our wind models were based on a relatively coarse DSM (60 m ground resolution). Given recent findings of the importance of local topography in modulating wind damage (Ibanez *et al*., 2024), it is possible that some localised wind sheltering effects might be better captured by TPI in the highly rugged landscape in the study area.

### Forest structural diversity created by lower resistance of wind‐sheltered forests against tropical cyclones

Interestingly, wind‐sheltered forests were found to be more susceptible to extreme TCs. Previous studies have reported conflicting evidence on whether forests in wind‐exposed locations were more resistant to TCs. In New Zealand, forests on leeward slopes were found to suffer more wind damage than their windward counterparts (Martin & Ogden, 2006). Similarly, both Weaver (1986) and Scatena & Lugo (1995) reported more windthrow in valleys and lowlands in hurricane‐prone forests in Puerto Rico. On the other hand, Bellingham (1991) observed heavy damage amongst forests on ridges in Jamaica after Hurricane Gilbert; Ostertag *et al*. (2005) found forests on ridges and valleys to suffer more heavily than those on slopes when Hurricane Georges hit Puerto Rico; and Yap *et al*. (2016) noted higher tree mortality only on windward slope and ridges after Category 4 Typhoon Imbudo hit Philippines. These seemingly conflicting results from previous studies likely stems from the tangled effects of several confounding factors. Estimating wind exposure by aspect, elevation, or topographical position tends to mix up the effects of forest height, long‐term mean wind speed, and exposure to the specific TC in question. The sample sizes of field‐based studies were also insufficient to resolve the effects of all these factors with confidence. Our study represents the first attempt to combine CFD wind modelling with large repeated LiDAR datasets. By studying forests across > 190 000 sites, we lifted many traditional limitations and conclusively showed that, amongst forests of the same height, those in sheltered sites were more susceptible to tropical cyclones (Figs 6, 8). While this may seem counterintuitive as these sheltered forests generally experience lower maximum wind speeds during tropical cyclones (Fig. 4), the pattern can be explained by wind acclimation. Forests regularly exposed to strong winds tended to change their species composition, canopy structure and tree architectures over time, which facilitated resistance to extreme TCs (Telewski & Jaffe, 1986; Bonnesoeur *et al*., 2016; Coomes *et al*., 2018; Jackson *et al*., 2019; Costa *et al*., 2023).

The two alternative wind‐acclimation strategies represent an underappreciated contributor to forest structural diversity. Forests topographically exposed to high‐mean wind speeds were structurally acclimated, TC‐resistant, and flat‐topped (Fig. 7). Contrarily, forests sheltered from wind were poorly wind acclimated and were rugged in structure due to gaps generated by TCs (Fig. 7). Previous work has shown that canopy structure and gap dynamics are tightly linked to floral and faunal compositions (Hector *et al*., 2011). For instance, rugged forests with many gaps facilitated the growth of lianas and changed butterfly composition in Borneo (Cleary *et al*., 2005). The three‐way interaction between wind, topography, and forest response may represent a crucial determinant of habitat structure, species composition, and beta diversity, which has been previously overlooked due to the lack of relevant data.

### Into the future: wind‐forest dynamics under a changing climate

Facing a changing climate, it is perhaps more important now than ever to study how strong winds during extreme TCs affect forests. Notably, our study highlights two aspects in wind‐forest dynamics that will be worth exploring in the future. First, plantations were found to be relatively ill‐adapted to extreme TCs (Fig. 9). This is concerning as fast‐growing plantations are widely used to meet carbon sequestration targets (Lewis *et al*., 2019). Their higher susceptibility to wind needs to be properly considered to ensure that restoration objectives are not undermined by wind disturbance under changing TC regimes. Second, our study demonstrated that the diverse wind patterns across rugged landscapes play a critical role in defining forest stature and structure in TC‐prone regions. The frequency of TCs in the west Pacific typhoon hotspot is currently on a downward trajectory and is projected to further decrease in the future (Knutson *et al*., 2010; Chand *et al*., 2022), while the intensity of TCs is expected to increase (Knutson *et al*., 2010; Kossin *et al*., 2020). These changes imply that forests would have fewer opportunities to wind‐acclimate during weaker TCs, leading to heavier losses in aboveground biomass when affected (Rau, 2022). In regions with rugged topographies, wind regimes tend to be highly local (Fig. 4) and associated forest structural variations will likely persist. Nevertheless, it will be valuable to further investigate the linkages between wind‐defined forest attributes and biodiversity metrics, which will allow us to gain a more thorough understanding of how changing wind regimes impact forest ecosystems.

## Competing interests

None declared.

## Author contributions

AHYC, TDJ and DAC planned and designed the research. AHYC and YKL collected wind data and generated plantation maps. AHYC analysed the data. AHYC, TDJ, E‐PR, and DAC interpreted the results. AHYC wrote the first draft of the manuscript. All authors edited the final draft of the manuscript.

## Disclaimer

The New Phytologist Foundation remains neutral with regard to jurisdictional claims in maps and in any institutional affiliations.

## Supporting information


**Fig. S1** Changes in heights of the canopy height model, digital surface model, and digital terrain model across different point densities of the LiDAR dataset.
**Fig. S2** The effects of lowering LiDAR point density on DSM heights.
**Fig. S3** Lowering the ground resolution of the DSMs by maximum resampling mitigates the drop in DSM due to lower LiDAR point densities.
**Fig. S4** The network of nonurban weather stations (*n* = 28) and our own anemometers (*n* = 8) across the complex topography of Hong Kong.
**Fig. S5** Predicted and actual long‐term mean wind speeds of 38 weather stations.
**Fig. S6** Predicted and actual mean wind speeds when typhoon or strong monsoon warnings were issued.
**Fig. S7** Correlation matrix between various environmental variables.
**Fig. S8** Standard diagnostic plots for the multiple regression model on canopy height changes between 2017 and 2020.
**Fig. S9** Semivariogram showing the spatial structure of the multiple regression model.
**Fig. S10** Density plot showing distribution of 2017 canopy heights amongst natural forests and plantations.
**Notes S1** Artefacts introduced by man‐made objects.
**Notes S2** Effects of point density on repeated LiDAR data.
**Notes S3** Wind modelling.
**Notes S4** Multiple regression model of 2017–2020 height change.
**Notes S5** Reweighting to compare forest resistance of plantations and natural forests.
**Table S1** Validating the wind models with our own anemometer measurements.
**Table S2** Summary statistics from the multiple regression model on 2017–2020 canopy height change.
**Table S3** Table showing the balance of covariates between natural forests and plantations before and after we reweighted the data.
Please note: Wiley is not responsible for the content or functionality of any Supporting Information supplied by the authors. Any queries (other than missing material) should be directed to the *New Phytologist* Central Office.

## Data Availability

The data that support the findings of this study are openly available in figshare at doi: 10.6084/m9.figshare.c.6940710.
