# Supplementary material for: Forest dynamics where typhoon winds blow
Source: New Phytol. 2024 Dec 14;245(6):2496–511. doi: 10.1111/nph.20350 (PMC11840412; doi:10.1111/nph.20350)
Supplement: Supplementary file 1 — Fig. S1 Changes in heights of the canopy height model, digital surface model, and digital terrain model across different point densities of the LiDAR dataset. Fig. S2 The effects of lowering LiDAR point density on DSM heights. Fig. S3 Lowering the ground resolution of the DSMs by maximum resampling mitigates the drop in DSM due to lower LiDAR point densities. Fig. S4 The network of nonurban weather stations (n = 28) and our own anemometers (n = 8) across the complex topography of Hong Kong. Fig. S5 Predicted and actual long‐term mean wind speeds of 38 weather stations. Fig. S6 Predicted and actual mean wind speeds when typhoon or strong monsoon warnings were issued. Fig. S7 Correlation matrix between various environmental variables. Fig. S8 Standard diagnostic plots for the multiple regression model on canopy height changes between 2017 and 2020. Fig. S9 Semivariogram showing the spatial structure of the multiple regression model. Fig. S10 Density plot showing distribution of 2017 canopy heights amongst natural forests and plantations. Notes S1 Artefacts introduced by man‐made objects. Notes S2 Effects of point density on repeated LiDAR data. Notes S3 Wind modelling. Notes S4 Multiple regression model of 2017–2020 height change. Notes S5 Reweighting to compare forest resistance of plantations and natural forests. Table S1 Validating the wind models with our own anemometer measurements. Table S2 Summary statistics from the multiple regression model on 2017–2020 canopy height change. Table S3 Table showing the balance of covariates between natural forests and plantations before and after we reweighted the data. Please note: Wiley is not responsible for the content or functionality of any Supporting Information supplied by the authors. Any queries (other than missing material) should be directed to the New Phytologist Central Office. [file NPH-245-2496-s001.docx]

## *New Phytologist* Supporting Information

Article title: Forest dynamics where typhoon winds blow

Authors: Aland H. Y. Chan, Toby D. Jackson, Ying Ki Law, E-Ping Rau, Billy C. H. Hau, David A. Coomes

Article acceptance date: 25 November 2024

The following Supporting Information is available for this article:

**Notes S1** Artifacts introduced by man-made objects

Man-made objects can confound products based on repeated LiDAR datasets used to assess changes in canopy height associated with typhoon damage and long-term growth. The high population density in Hong Kong means that man-made objects often encroach into the countryside. To ensure that our results were not affected by rural settlements, villages, and associated agricultural practices, we focused our analysis on areas of Hong Kong with minimal disturbance. Compared to most developing countries, land use regulations within protected areas are relatively well-enforced in Hong Kong, so we used the government zoning map to narrow down our study area to Country Parks, Green Belts, and Coastal Protected Areas (Town Planning Board, 2020). Within protected areas, power lines that traverse through the landscape cause additional issues in height change estimates. Power lines often lead to large changes in canopy heights when swayed by wind or when two LiDAR datasets were slightly spatially misaligned. To mitigate this issue, we extracted the locations of power lines from OpenStreetMap and masked out pixels within a 40m buffer (OpenStreetMap contributors, 2022).

**Notes S2** Effects of point density on repeated LiDAR data

The differences in LiDAR point densities between datasets represent another potential source of error in our analysis. In this section we present results from a sensitivity analysis where we test how LiDAR point densities affected DTM, CHM, and DSM construction. We conduced the study in the Mau Ping, Hong Kong, which includes vegetation ranging from short shrublands to mature secondary forests. The 2020 LiDAR dataset, which has the highest point density amongst the three datasets, was repeatedly thinned using the *lasthin* function in *LAStools*. More specifically, we lowered the point densities (pd) to 50, 30, 10, 7, 3, 2, 1, 0.5, and 0.25 points per m^2^. We then constructed DTMs, CHMs, and DSMs using the same functions described in the main text.

As a first step, we used the DTM, CHM, and DSM constructed from pd = 50m^2^ point cloud as a benchmark and measured how heights changed when the pd was lowered. The results are presented in **Figure S1**. Overall, the lower the point density, the smaller the chance for some of the points to hit the ground layer, so the DTM gets overestimated (**Figure S1**). In contrast, a sparser point cloud makes it difficult to capture the tops of trees, so DSMs tend to be underestimated (**Figure S1**). CHM suffers from the largest drop in height as point density drops, as it is sensitive to both the overestimation of the ground layer and the underestimation of top of canopy height (**Figure S1**). The results support our use of the DSM in estimating height changes as it was more robust to variations in point density.
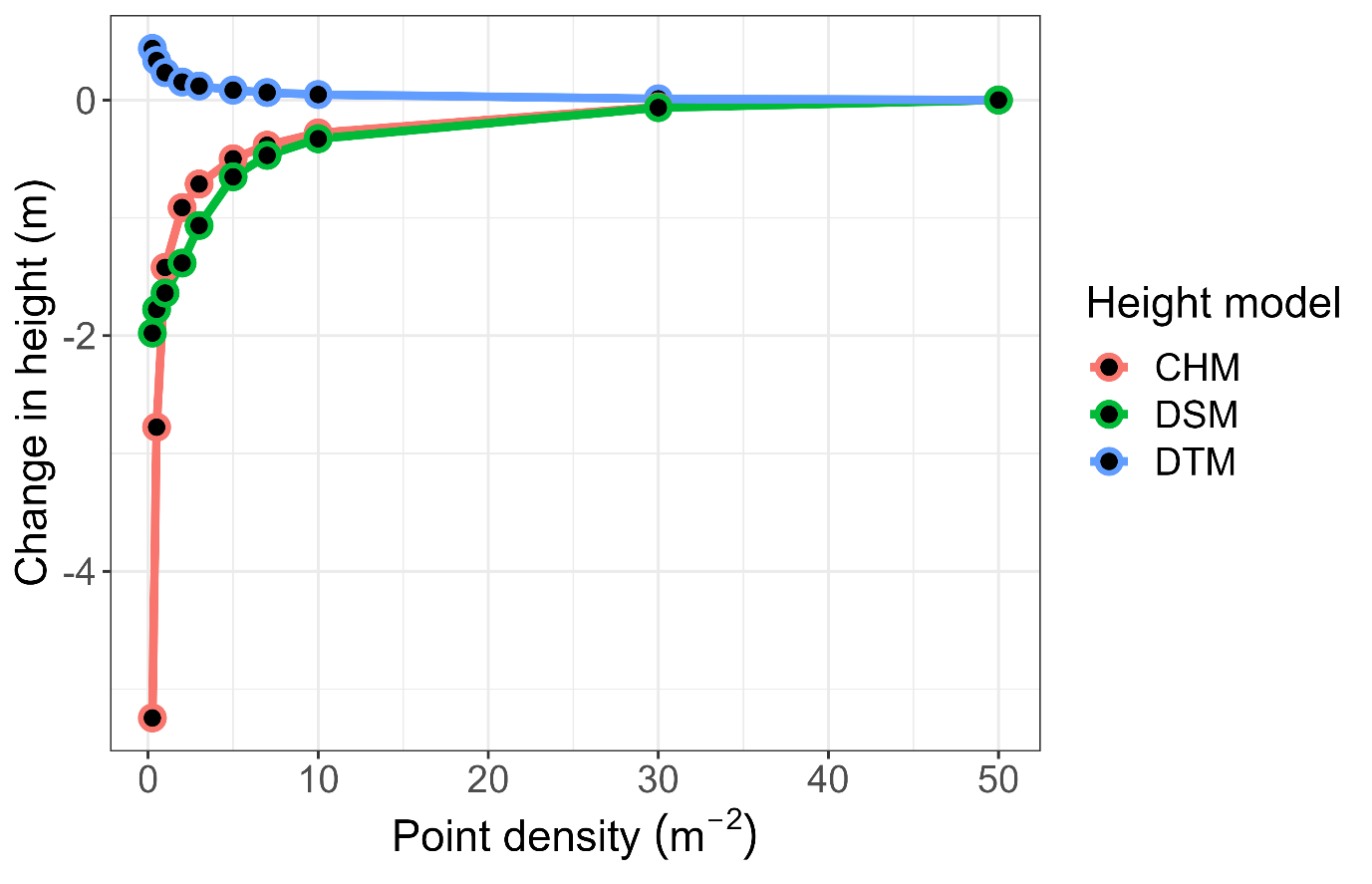


**Fig. S1** Changes in heights of the canopy height model (CHM), digital surface model (DSM), and digital terrain model (DTM) across different point densities of the LiDAR dataset. We used the CHM, DSM, and DTM estimated from the pd = 50 dataset as the benchmark to calculate changes in height as we lowered the point density.

Secondly, we tested whether the strength of point density effects correlated with forest height. As expected, given the same point density, the heights of taller forests tend to be underestimated more. The magnitude of this point density effect was, however, manageable as long as point densities do not fall too low. For all height classes, the errors were <1m when pd = 2 and <0.5m when pd = 3.

Part of these errors could be mitigated by lowering the resolution of the DSMs. The results from **Figure S2** represent the errors of the original DSM with 1 m ground resolution. If the DSMs were resampled to a larger pixel size by taking the maximum, there might be a better chance of capturing the tops of trees. **Figure S3** demonstrates how increasing the ground resolution from 1 m to 2-5 m could reduce the effects of low point densities on DSM height estimates.


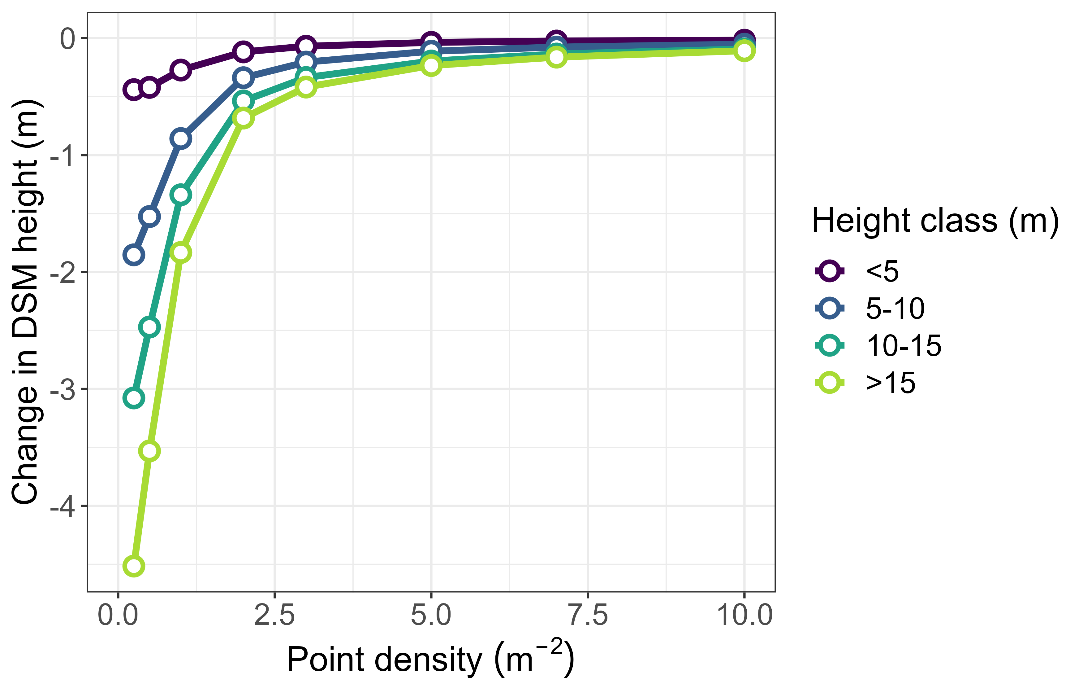


**Fig. S2** The effects of lowering LiDAR point density on DSM heights. The DSM generated from the pd = 50 m^-2^ point cloud was used as the benchmark to calculated height differences. The four lines represent pixels of different canopy heights.


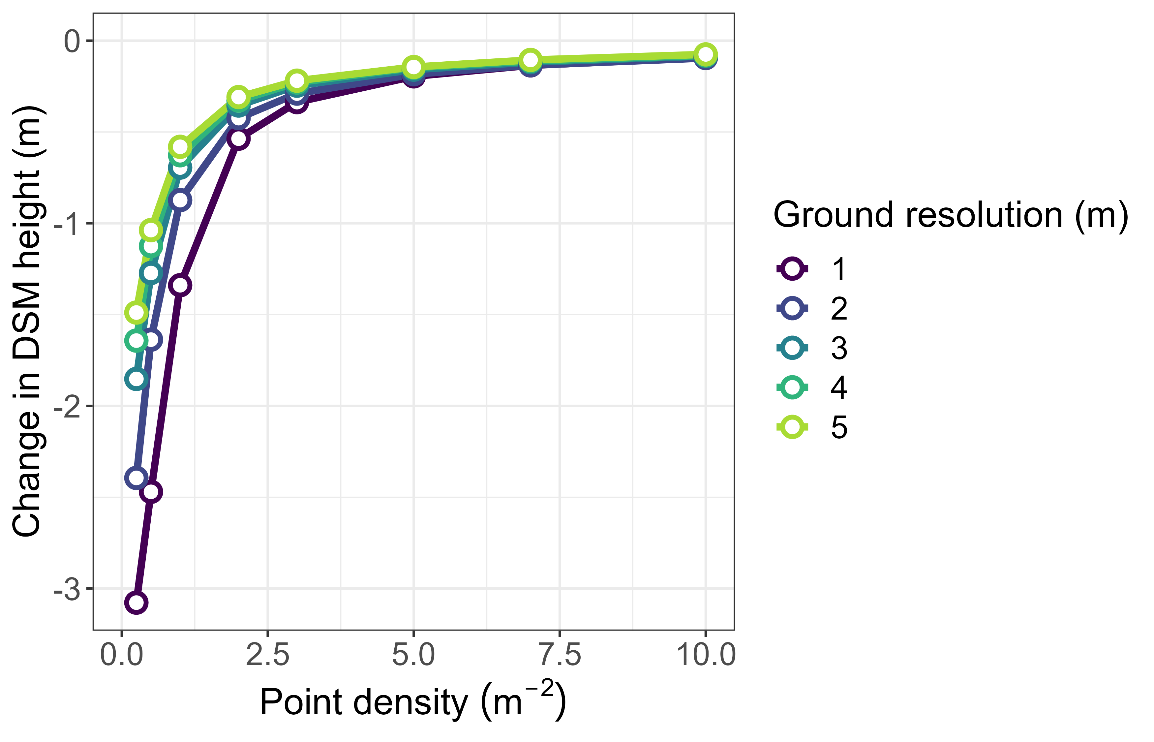


**Fig. S3** Lowering the ground resolution of the DSMs by maximum resampling mitigates the drop in DSM due to lower LiDAR point densities. This graph represents trees in the 10-15 m height class. The DSM created from the pd = 50 m^2^ was used as a benchmark.

In light of the results from the sensitivity analysis, we adjusted our methodology in estimating changes in canopy height. Firstly, we differenced the DSMs instead of the CHMs as DSMs were less sensitive to changes in point density. Secondly, we lowered the point density of the 2020 dataset to 5.4 m^-2^ to match that of the two other datasets. Thirdly, when relevant, we excluded regions that had a point densities <1.5 m^-2^ from the 2017 dataset, which should control the errors of the DSM to <1 m. Fourthly, to further mitigate these errors, we resampled the DSMs to 2 m ground resolution before differencing DSMs to estimate canopy height change. Lastly, it is important to note that errors in **Figures S1-S3** were calculated by comparing the DSMs with another DSM created from the pd = 50 m^-2^ point cloud. In our actual analysis, we were differencing DSMs created from similar point densities (i.e. all datasets would have been slightly underestimating height). After differencing, these errors would have partially been cancelled out. Hence, in the 2010-2017-2020 height change dataset, the actual errors attributable to differences in point density would be substantially smaller than that presented in **Figures S1-S3**.

**Notes S3** Wind modelling

*Overview*

In this section we will provide a more detailed description of the wind modelling pipeline. As outlined in the main text, the purpose of modelling wind flows is to create two products - (1) a raster showing long-term mean wind speed and (2) a raster showing maximum wind speed during Typhoon Mangkhut. We will start the section by describing the datasets used for wind modelling and product validation. This is followed by a detailed description on how we built the wind model. Finally, we will present results from the wind model validation exercise.

*Datasets used for wind modelling and validation*

The background topography of Hong Kong was mapped using a LiDAR-derived digital surface model (DSM) as described in the main text. In particular, we used the 2020 dataset as it has the largest coverage and highest point density (54 points/m^2^) amongst the three repeated LiDAR datasets. Since mountains located outside Hong Kong could affect wind speeds in the territory, we further expanded the 2020 LiDAR-based DSM using SRTM Digital Elevation Data Version 4 (Jarvis *et al.*, 2008). Specifically, we added a 15 km buffer to the existing DSM to include parts of Shenzhen, including Wutong Mountain (943.7 m.a.s.l.) and the Dapeng Peninsula.

Wind data used to build and validate the model were obtained from the Hong Kong Observatory. The Hong Kong Observatory manages a network of 38 anemometers stationed on automatic weather stations across the study area. The dataset we used spanned 37 years (1984-2022) and consists of hourly wind speed, wind direction, and gust records. An issue with wind data collected in Hong Kong is the presence of high rising buildings in urban areas. We therefore removed 10 stations that were within 500m of tall buildings and skyscrapers, leaving data collected from 28 non-urban weather stations for wind modelling. Natural forests and plantations have a scattered distribution across the territory and are often inter-mixed (Abbas, Nichol and Fischer, 2016; Chan and Coomes, 2024). Hence, data from these weather stations are representative of conditions experienced by both studied forest types in Hong Kong.

We collected additional wind data from temporary cup anemometers on mountain slopes in Hong Kong. While the 28 non-urban weather stations represent one of the densest local anemometer networks in TC-prone regions, they are mainly located on mountaintops, rooftops of man-made structures, or near the coast. To ensure that our wind models accurately estimate wind speeds on slopes, we devised a temporary cup anemometer linked to a HOBO U30 data logger. The anemometer was moved around between eight hillslope sites. The sites were chosen such that the surrounding area was devoid of tall vegetation to avoid local wind shadows cast by trees. The anemometer was secured on a plastic pipe mounted on a tripod at a height of 2.4 m aboveground and measures wind speed and gusts every 30 seconds. The locations where wind data was collected are shown in **Figure S4**.


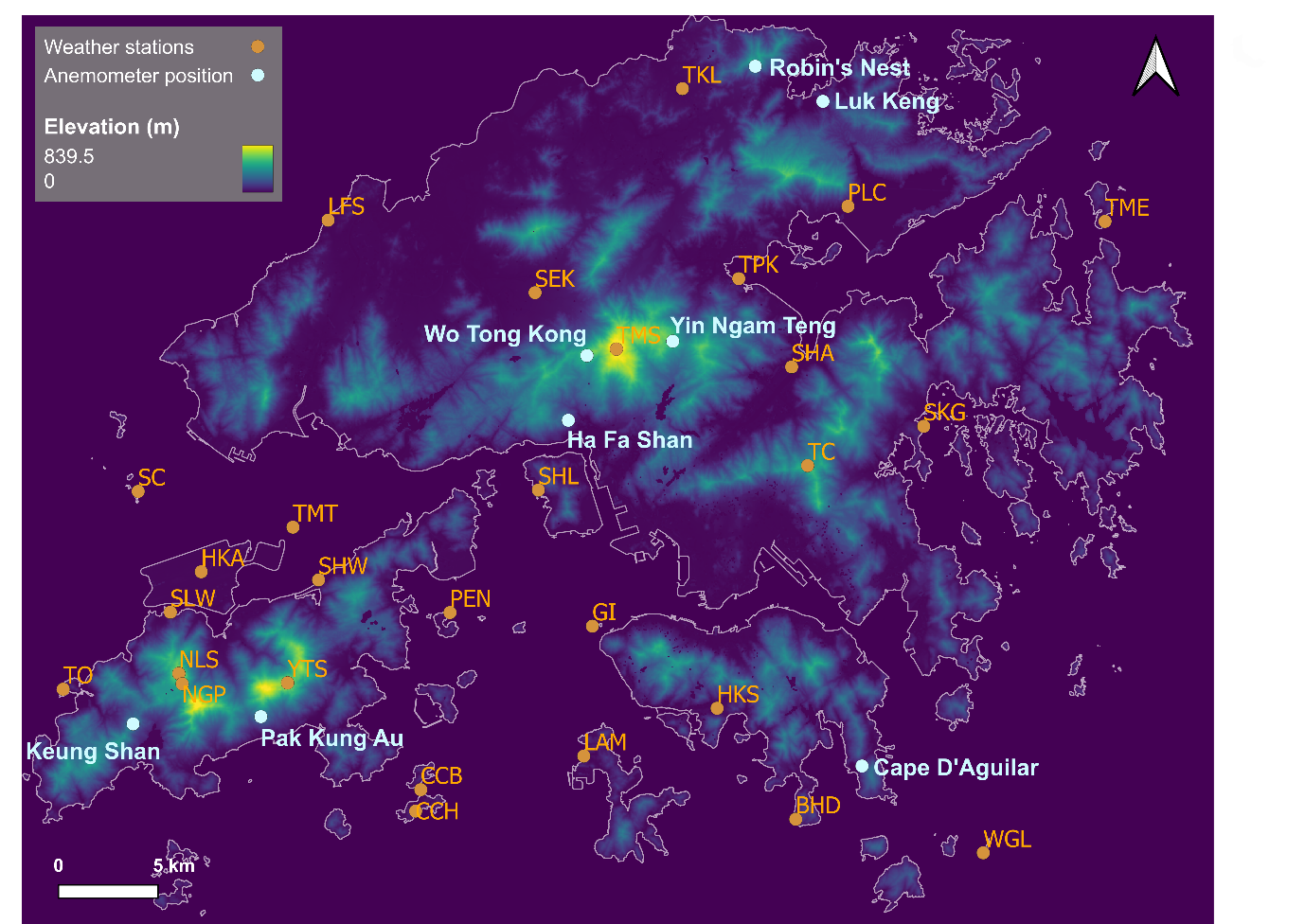


**Fig. S4** The network of non-urban weather stations (n = 28) and our own anemometers (n = 8) across the complex topography of Hong Kong. The code names of the weather stations refers to that used by the Hong Kong Observatory (Hong Kong Observatory, 2023). The background shows the LiDAR-derived digital terrain model of Hong Kong with the land area being outlined.

*Building the wind model*

We modelled surface winds in Hong Kong by combining outputs of the “conservation of mass and momentum solver” in WindNinja. WindNinja (available on https://weather.firelab.org/windninja/) is a free CFD modelling software developed by the Missoula Fire Sciences Laboratory (Forthofer et al., 2014). The programme provides solvers to estimate wind speeds across the landscape at user-specified heights above ground based on two inputs – (1) a digital surface model (DSM) of the area and (2) a set of initial conditions (domain average wind speed and direction). Two separate solvers are available in WindNinja – the “conservation of mass” and the “conservation of mass and momentum” solver. While the former is designed to maximise computational speed for wildfire behaviour modelling, we would focus on the latter as previous validation efforts have found it to provide more realistic approximations of surface winds, especially on leeward slopes in complex terrains (Wagenbrenner et al., 2019). The “conservation of mass and momentum” solver is a numerical model based on Reynolds-Averaged Navier-Stokes (RANS) equations, assuming steady, incompressible, turbulent, and neutrally-stratified wind flows. Its mesh creation and CFD calculations are built upon OpenFOAM, a free and open-sourced CFD modelling software (Jasak, 2009; Wagenbrenner et al., 2019).

Using WindNinja, we modelled wind speeds for 128 domain average wind scenarios. Specifically, we generated wind speed and direction rasters from the products of 8 compass directions (0^o^, 45^o^, 90^o^, 135^o^, 180^o^, 225^o^, 270^o^, 315^o^) and 16 wind speeds in km/h (1, 8, 13, 17, 21, 24, 28, 32, 37, 44, 50, 70, 100, 150, 200, 250). The mesh resolution was set to 60 m; background vegetation type was set to “-brush”; and both the input and output wind height was set to 10 m aboveground to match that of the anemometer data. The modelling was carried out in a Windows workstation with 48 cores and 1TB RAM.

We then used combinations of the 128 domain average wind scenarios to estimate time-resolved surface wind speeds. In order to cross-validate the results, we first split the wind data collected from the 28 weather stations into 10 folds. In each of the 10 iterations, we took the wind measurements from 9 folds as training and used the remaining fold for validation. During every hour in the 37 years for which we have data, we identified the four scenarios where wind directions and speeds most closely matched that of the training data. We then took a weighted average of the four scenarios to obtain a time-specific estimation of surface wind direction and speed. As a demonstrative example, if we observed winds blowing from 30^o^ NNE at 9 km/h, we take wind speed rasters of the four corresponding scenarios (0^o^-8km/h, 0^o^-13km/h, 45^o^-8km/h, 45^o^-13km/h), weight them according to how close the scenario was to observed conditions (30^o^-9km/h), then average the weighted rasters to get final estimated wind speed. WindNinja currently does not incorporate variations in surface roughness in its CFD model, which makes it prone to overestimating wind from rough urban areas and underestimating wind from smoother oceans. Hence, we applied a simple roughness correction on the modelled outputs. We assigned roughness lengths to pixels based on its land cover class following Wieringa (1986). For each pixel, we then estimated directional roughness by calculating distance-weighted roughness of the eight compass directions. We then corrected the estimated wind speeds using a simple linear regression model (prediction error ~ roughness + roughness:predicted wind speed). The accuracy of the roughness-corrected wind speed estimates was validated using the holdout validation dataset.

Alternatively, we built a null model where we used (1) the mean wind speed across all stations and (2) elevation to predict location-specific wind speeds. We compared the performance of the CFD-based model and the null model in predicting (1) overall mean wind speeds of weather stations and (2) wind speeds during typhoons or monsoons. The accuracies of the wind models were evaluated by calculating the absolute root mean square error (RMSE) and percentage root mean square error (%RMSE) between the actual and predicted wind speed.

Finally, apart from cross validating the wind models using the weather station data, we also validated the models with our own anemometer measurements on slopes. We used the weather station data to produce time-resolved hourly estimates of surface wind speeds at the locations where we set up our own anemometer. The estimated surface wind speed, both by the CFD model and the null model, were compared to the wind speeds we observed at the site. It is important to note that our anemometers were measuring wind speed at approximately 2.4 m above ground. Although wind speeds 2.4 m above ground (observed) would be correlated with speeds at 10 m above ground (model prediction), local surface roughness caused by vegetation could easily introduce location-specific, systematic biases. Hence, RMSE and %RMSE is not a good measurement of model accuracy. We therefore used an alternative way to validate the models and relied on the R2 of linear regression models through the data (actual ~ predicted wind speeds) instead.

*Validating the wind models*

The wind model derived from computational fluid dynamics (CFD) modelling performed better than the null model derived from elevation and mean wind speed across all stations. We started by validating long-term mean wind speed predictions. Both the CFD model and the null model were used to predict the long-term mean wind speed of the 38 non-urban weather stations (10-fold cross validation). The root mean square error (RMSE) of the CFD model was 3.77 km/h, representing a percentage RMSE (%RMSE) of 30%. Meanwhile, the null model had a higher RMSE of 4.22 km/h, representing a %RMSE of 41%. The results are summarised in **Figure S5**, where the vertices of each polygon represent the predicted and actual mean wind speeds for wind blowing from the eight compass directions. An ideal wind model should produce points close to blue line, with edges of polygons parallel to the blue line. While elevation does capture some variation of wind speed, the null model was making predictions at the 10-15 km/h range for most weather stations at low elevations. The CFD model, being able to capture the effects of wind shadows, produce more reasonable estimates that line up better with the blue 1:1 line. Errors of the CFD model were mainly from two sources. (1) Many weather stations were located on ridges, which often represents the border between the windward and leeward slopes. The model occasionally places the weather stations on the wrong side of the border, leading to large over- or under-predictions. (2) Variations in surface roughness was not incorporated in the CFD model, and our correction only partially mitigated the issue. The former issue is probably less important as small differences in the position of the wind shadows are unlikely to affect subsequent analysis. The latter issue is worth addressing in future work.

As we are interested in modelling the effects of Typhoon Mangkhut, we carried out a separate cross validation exercise for high wind speed scenarios. We found that the CFD model performs better when a typhoon or strong monsoon warning was issued by the Hong Kong Observatory (**Figure S6**). With higher wind speeds, the RMSE was slightly higher (4.22 km/h), but the %RMSE dropped to 22%. Under these scenarios, the advantages of the CFD model over the null model were also more apparent. The null model generated many near-vertical polygons far from parallel with the 1:1 line (**Figure S6**). This is likely due to mountains casting bigger and clearer wind shadows under strong wind.


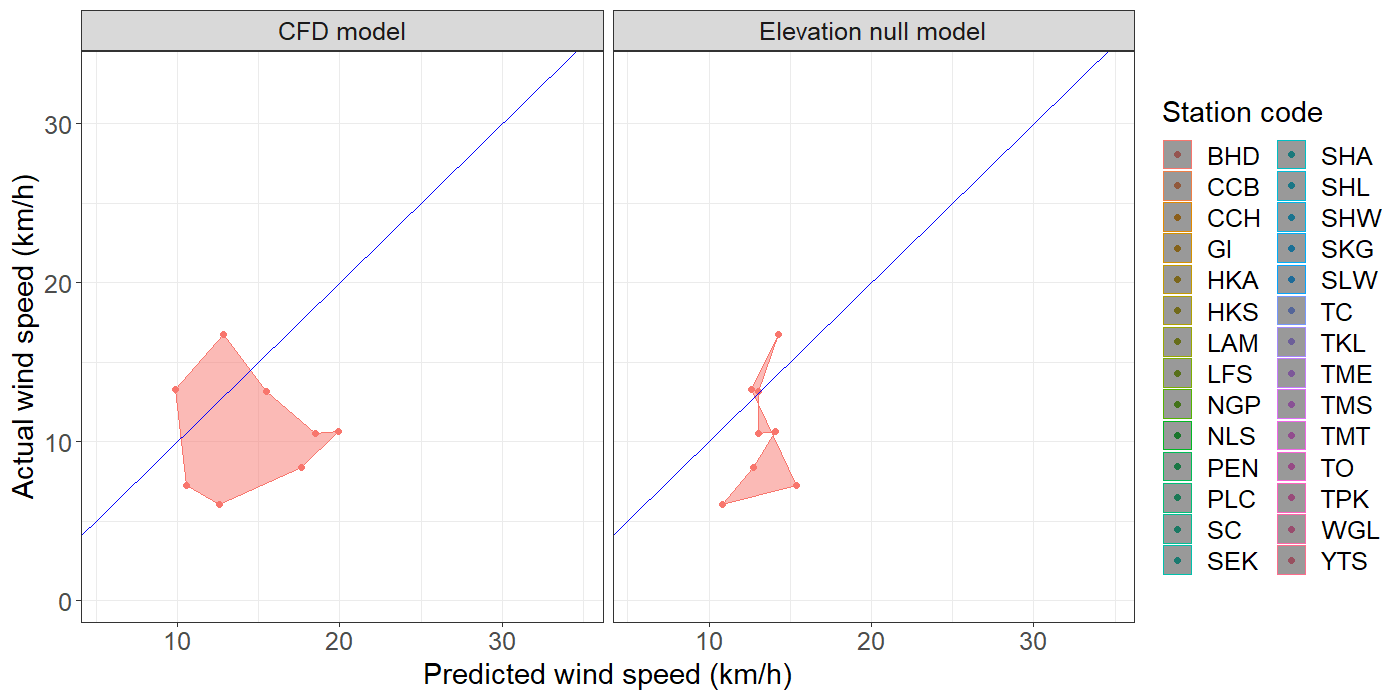


**Fig. S5** Predicted and actual long-term mean wind speeds of 38 weather stations. Each point represents wind approaching from one of the eight compass directions. The blue line indicates perfect prediction.


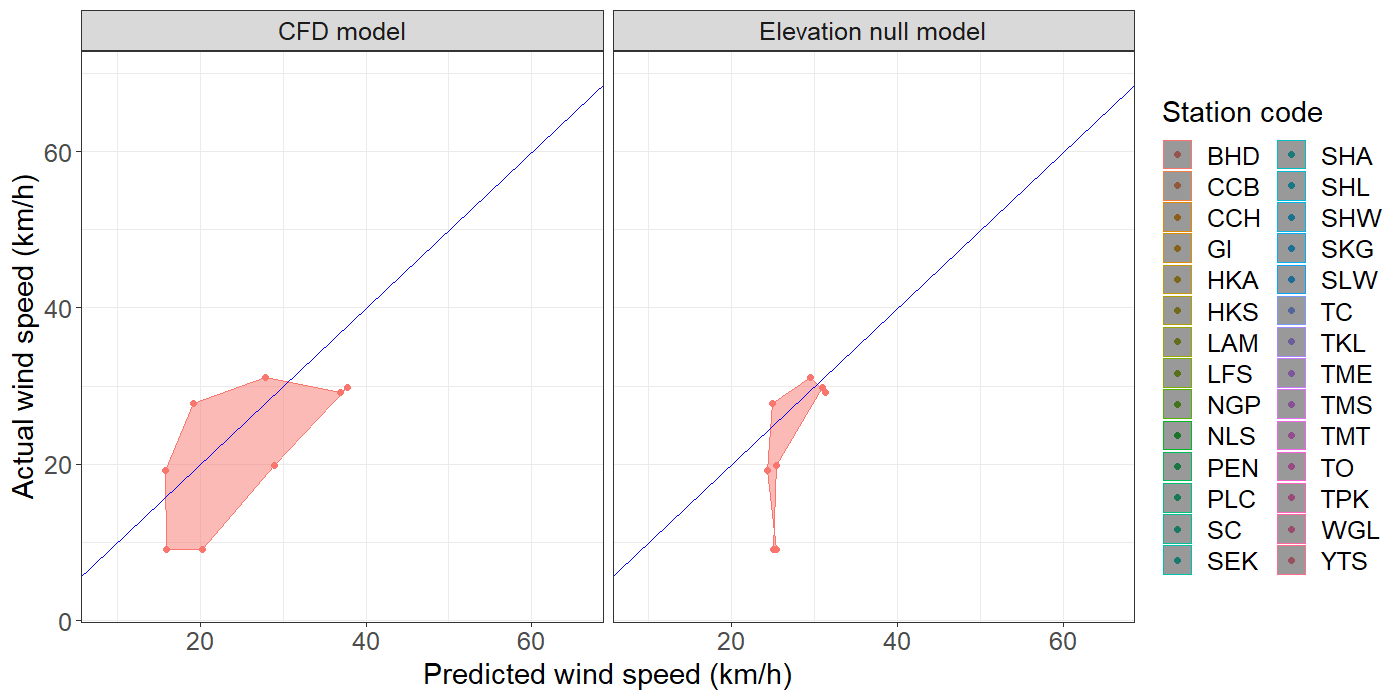


**Fig. S6** Predicted and actual mean wind speeds when typhoon or strong monsoon warnings were issued. The coloured polygons represent 38 non-urban weather stations. Each point represents wind approaching from one of the eight compass directions. The blue line indicates perfect prediction.

Finally, we also validated our wind model using data from our own anemometer readings at eight locations. We fitted linear models between predicted and actual wind speeds. The R^2^ values of these linear models are tabled in Table S1. Overall, the performance of the CFD model was similar to the null model that used mean wind speed and elevation as predictors. This is likely because the sites were not very windy during the study period. Wind shadow and speedup due to mountains were probably not particularly important during calm conditions. We did qualitatively observe that the CFD model tend to outperform the null model during winder days of measurements (Wo Tong Kong, Keung Shan, and Cape D’Aguilar), but the sample size is too small for any meaningful statistical tests. It is also important to note that we only had 4 hours of data from Robin’s Nest, and the Luk Keng site was partially occluded by trees, but we still included the data here for completeness.

**Table S1** Validating the wind models with our own anemometer measurements. We built linear models for actual wind speed against predicted wind speed. Higher R2 values indicates better agreement between model predictions and actual wind speed. Mean wind represents mean wind speed across all measurements from that anemometer. Duration represents how long the anemometer had been place out.

| Location | Duration (h) | Mean wind (km/h) | CFD model R^2^ | Null model R^2^ |
| --- | --- | --- | --- | --- |
| Wo Tong Kong | 73 | 13.7 | **0.23** | 0.17 |
| Keung Shan | 116 | 11.6 | **0.45** | 0.40 |
| Cape D’Aguilar | 77 | 9.0 | **0.80** | 0.75 |
| Robin’s Nest | 4 | 7.5 | -0.47 | **0.94** |
| Pak Kung Au | 30 | 7.1 | 0.06 | **0.13** |
| Ha Fa Shan | 23 | 7.0 | 0.81 | **0.85** |
| Yin Ngam Teng | 21 | 5.8 | **0.70** | 0.30 |
| Luk Keng | 28 | 0.7 | 0.08 | **0.10** |

**Notes S4** Multiple regression model of 2017 – 2020 height change

In the main text we described a multiple regression model that use different variables to predict canopy height change between 2017 and 2020 (damage by Typhoon Mangkhut). Here we (1) provide additional information on the variable selection process and (2) detail the results of the model.

A number of environmental variables relevant to typhoon damage were measured or estimated in the study, namely 2017 canopy height, aspect, cosine aspect, elevation, mean wind speed, maximum Mangkhut wind speed, normalised maximum Mangkhut wind speed, Saga wetness index (SWI), topographical position index (TPI), and slope. Amongst these variables, elevation and aspect were used to build the wind models. Aspect additionally had a cyclical effect on forests and its linearised form (cosine aspect) would have complicated correlations with the wind variables. Hence, we excluded elevation, aspect, and cosine aspect from the model. We then generated a correlation matrix with the remaining variables (**Figure S7**). We observed that long-term mean wind speed had a strong correlation with maximum wind speed during Typhoon Mangkhut (**Figure S7**). Hence, instead of using both variables in the model, we predicted maximum wind speed from the long-term mean wind speed, then subtracted these predicted values from the maximum Mangkhut wind speed to create a normalised variable (norm. max). The new variable was more orthogonal to long-term mean wind speed. The three remaining topographical variables (SWI, TPI, and slope) were also somewhat correlated with each other (**Figure S7**). We built the models with different permutations of these topographical variables and found that TPI had relatively small effect sizes on its own but significantly affected the coefficients of the other two variables. Hence, we built the final model without TPI. The resulting coefficients and the variance inflation factors (VIFs) for all variables in the final model could be found in **Table S2**.


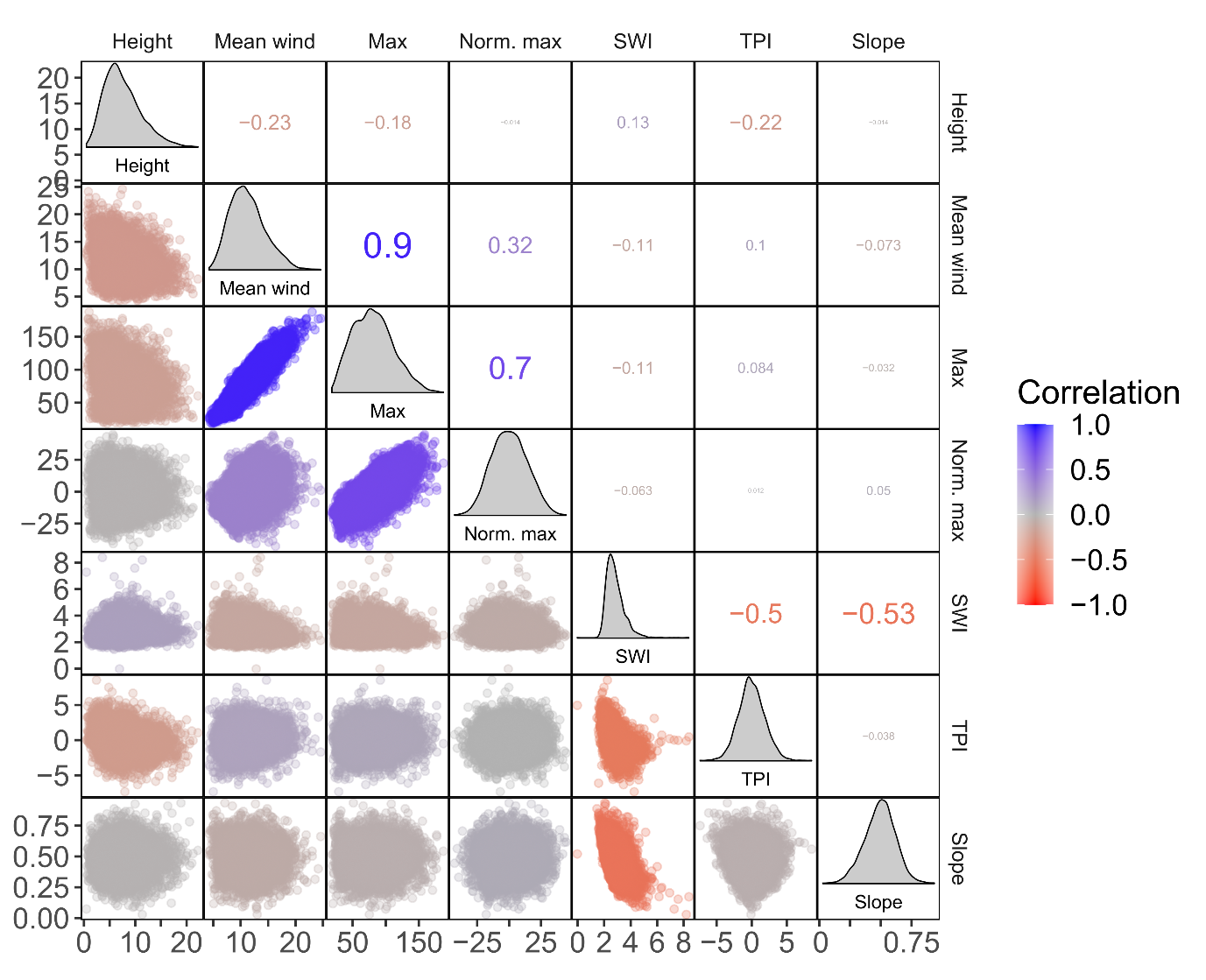


**Fig. S7** Correlation matrix between various environmental variables

**Table S2** Summary statistics from the multiple regression model on 2017 – 2020 canopy height change. The sample size was 191704 pixels, each 30 m by 30 m in size. VIF = Variance Inflation Factor.

| Variable | Estimate | Std. error | *t* value | *p*-value | VIF |
| --- | --- | --- | --- | --- | --- |
| (Intercept) | -0.091 | 0.0016 | -57.8 | <0.0001 | N/A |
| 2017 canopy height | -0.212 | 0.0015 | -138.5 | <0.0001 | 1.10 |
| Mean wind | 0.033 | 0.0016 | 20.5 | <0.0001 | 1.25 |
| Norm. max wind | -0.054 | 0.0016 | -34.7 | <0.0001 | 1.17 |
| Height : Mean wind | 0.035 | 0.0016 | 22.6 | <0.0001 | 1.18 |
| Height : Norm. max wind | -0.020 | 0.0016 | -12.5 | <0.0001 | 1.18 |
| Mean : Norm. max wind | 0.020 | 0.0016 | 12.3 | <0.0001 | 1.07 |
| SWI | -0.036 | 0.0018 | -20.6 | <0.0001 | 1.48 |
| Slope | 0.056 | 0.0018 | 31.7 | <0.0001 | 1.45 |

We also noted that multiple regression models run on several assumptions of normality, homoscedasticity, and independence of data points. Standard diagnostic plots for the model can be found in **Figure S8**. Notably, the dataset showed a higher frequency of extreme values than expected by the assumption of normality. This is mainly due to some large height changes in areas where the typhoon created large gaps that is comparable or larger than the pixel size. For predictive modelling, this would potentially be an issue that needs to be solved by increasing the pixel size. In this study, the model was used for evaluating the relative importance of the different variables in relation to forest resistance to extreme TCs, which should be robust against this skew. The second issue surrounds spatial autocorrelation - the phenomenon where pixels closer to each other are expected to be more similar to each other (non-independent). To assess the spatial structure of the model, we constructed a diagnostic semivariogram for the model using the *nmle* package in *R* (**Figure S9**). A subsampled dataset (*n* = 10000) was used to circumvent computational limitations. We observed some spatial autocorrelation for distances <7km, but the semivariance was overall high at relevant spatial scales of the study. Given the size of the dataset, apart from a slight underestimation of standard errors, we believe that spatial autocorrelation would not change the effect sizes and significance of the model. Therefore, we decided to keep the existing model architecture.

**
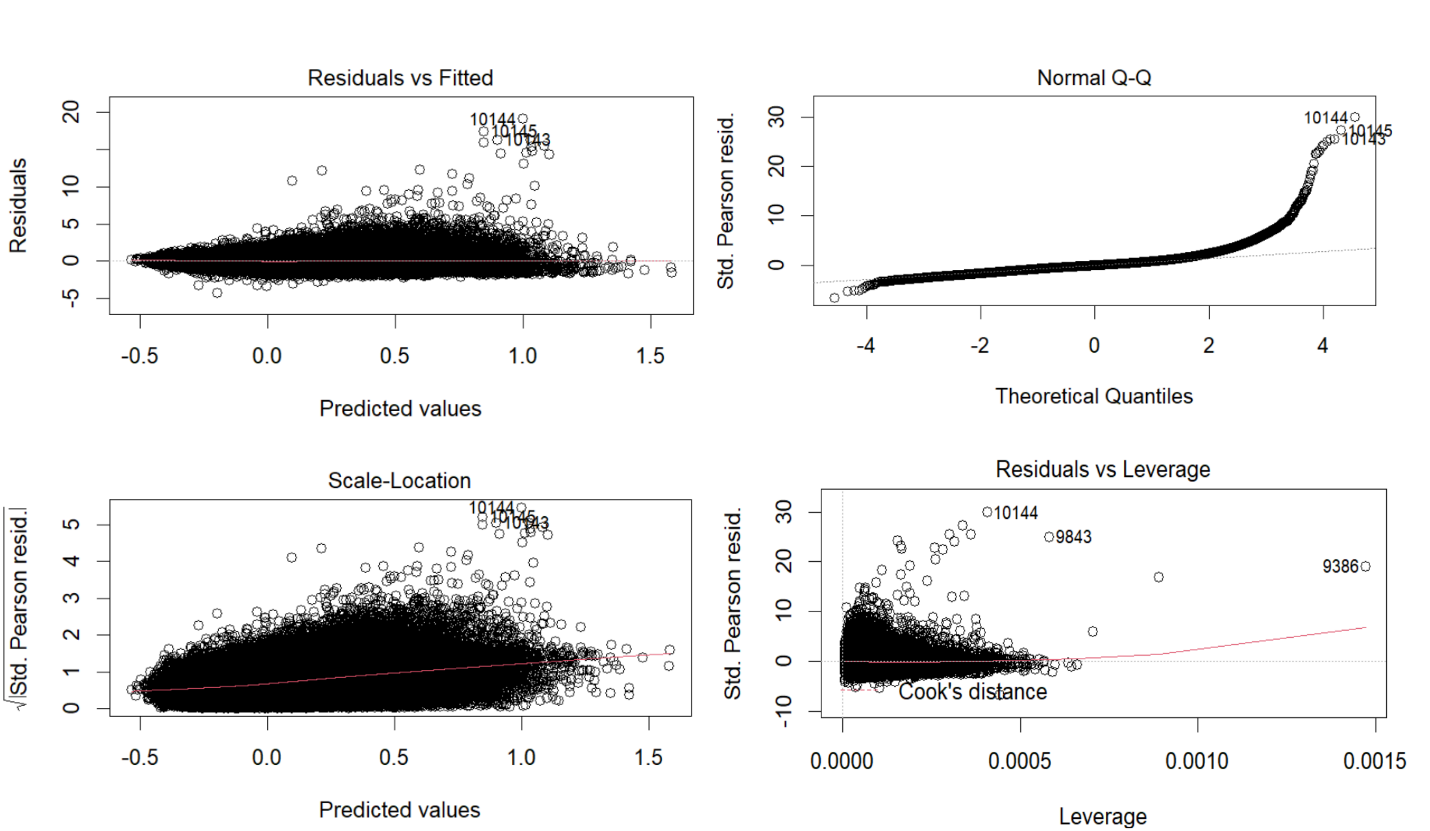
**

**Fig. S8** Standard diagnostic plots for the multiple regression model on canopy height changes between 2017 and 2020.


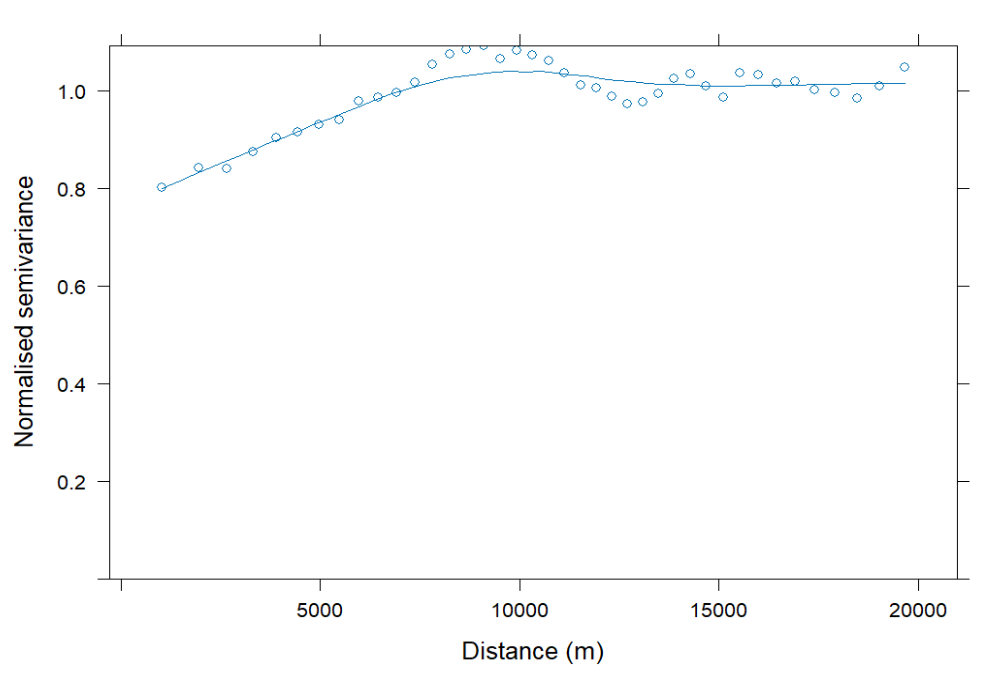


**Fig. S9** Semivariogram showing the spatial structure of the multiple regression model. The plot is generated from a subsampled dataset (*n* = 10000) due to computational limitations.

**Notes S5** Reweighting to compare forest resistance of plantations and natural forests

In this section we describe the steps we took to tackle covariate imbalance between natural forests and plantations before their resistances to strong typhoons. We followed the reweighting protocol described by Markoulidakis et al. (2022). Firstly, we removed parts of the dataset with little overlap (e.g. tall plantations >23 m that had insufficient analogous natural forest pixels for meaningful comparison) (**Figure S10**). Secondly, entropy balancing (EBAL) weights were assigned to the data using the *WeightIt* package in *R* (Greifer, 2019) based on the five covariates. Thirdly, the weights were trimmed at the 99.9^th^ percentile such that results were not overwhelmed by several heavily weighted pixels. Fourthly, the *cobalt* package was used to confirm that, after weighting, plantation and natural forest pixels had comparable distributions of covariates (mean differences <0.05 and variance ratios <2) (**Table S3**) (Greifer, 2020). The weighted heights were then used to conduct a like-for-like comparison of typhoon-resistance between natural and planted forests.


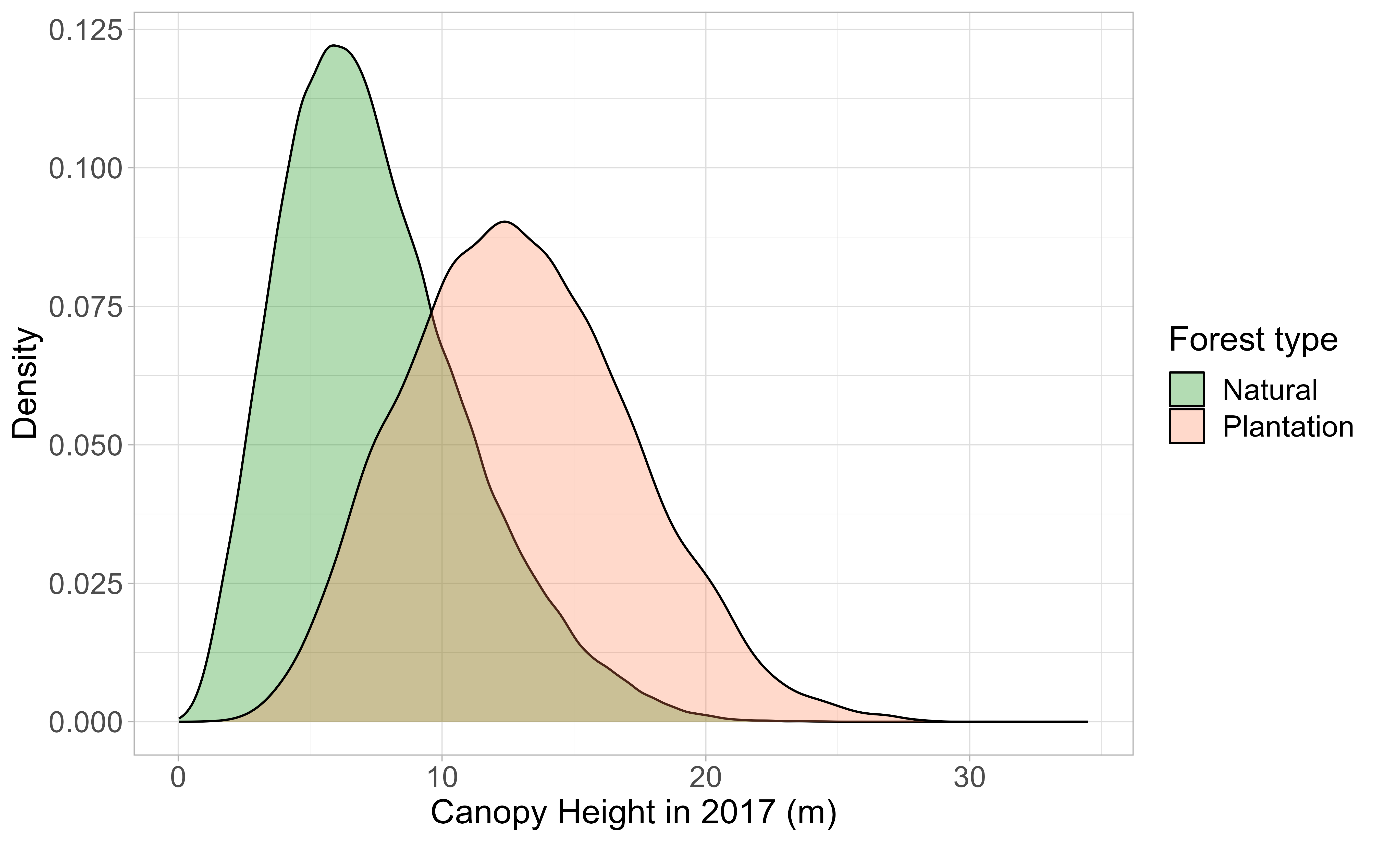


**Fig. S10** Density plot showing distribution of 2017 canopy heights amongst natural forests and plantations. Pixels with heights little overlap (<3 m and >23 m) were removed before reweighting to prevent overly large weights and unfair comparisons.

**Table S3** Table showing the balance of covariates between natural forests and plantations before and after we reweighted the data. Covariates are considered balanced when standardised mean differences are <0.05. Additionally, variance ratios close to 1 are preferred. See Greifer (2020) for more details.

| Variable | Before reweighting | | After reweighting | |
| --- | --- | --- | --- | --- |
|  | Mean difference | Variance ratio | Mean difference | Variance ratio |
| TPI | 0.36 | 0.86 | 0.01 | 0.88 |
| SWI | -0.17 | 0.74 | -0.01 | 0.80 |
| Mean wind | 0.21 | 1.13 | 0.01 | 0.95 |
| Max wind | 0.09 | 1.10 | 0.00 | 1.10 |
| Canopy height 2017 | 1.21 | 1.52 | 0.03 | 0.93 |

**References**

Abbas, S., Nichol, J. E. and Fischer, G. A. (2016) ‘A 70-year perspective on tropical forest regeneration’, *Science of the Total Environment*. Elsevier B.V., 544, pp. 544–552. doi: 10.1016/j.scitotenv.2015.11.171.

Chan, A. H. Y. and Coomes, D. A. (2024) ‘Fire traps in the wet subtropics: New perspectives from Hong Kong’, *Journal of Applied Ecology*. John Wiley & Sons, Ltd, 61(4), pp. 884–898. doi: 10.1111/1365-2664.14575.

Greifer, N. (2019) ‘Package “WeightIt”’. CRAN.

Greifer, N. (2020) ‘Covariate balance tables and plots: a guide to the cobalt package’, *Accessed March*, 10, p. 2020.

Jarvis, A. *et al.* (2008) *Hole-filled SRTM for the globe Version 4, available from the CGIAR-CSI SRTM 90m*. Available at: https://srtm.csi.cgiar.org.

Markoulidakis, A. *et al.* (2022) ‘A tutorial comparing different covariate balancing methods with an application evaluating the causal effects of substance use treatment programs for adolescents’, *Health Services and Outcomes Research Methodology*. Springer, pp. 1–34. doi: 10.1007/S10742-022-00280-0/FIGURES/3.

OpenStreetMap contributors (2022) ‘Planet dump retrieved from https://planet.osm.org’.

Town Planning Board (2020) ‘Town Planning Board, Hong Kong’. Available at: https://www.pland.gov.hk/pland_en/info_serv/digital_planning_data/download.htm.

Wieringa, J. (1986) ‘Roughness-dependent geographical interpolation of surface wind speed averages’, *Quarterly Journal of the Royal Meteorological Society*. John Wiley & Sons, Ltd, 112(473), pp. 867–889. doi: 10.1002/QJ.49711247316.
